# Testing times: trends in availability, price, and market share of malaria diagnostics in the public and private healthcare sector across eight sub-Saharan African countries from 2009 to 2015

**DOI:** 10.1186/s12936-017-1829-5

**Published:** 2017-05-19

**Authors:** ACTwatch Group, Louis Akulayi, Angela Alum, Andrew Andrada, Julie Archer, Ekundayo D. Arogundade, Erick Auko, Abdul R. Badru, Katie Bates, Paul Bouanchaud, Meghan Bruce, Katia Bruxvoort, Peter Buyungo, Angela Camilleri, Emily D. Carter, Steven Chapman, Nikki Charman, Desmond Chavasse, Robyn Cyr, Kevin Duff, Gylsain Guedegbe, Keith Esch, Illah Evance, Anna Fulton, Hellen Gataaka, Tarryn Haslam, Emily Harris, Christine Hong, Catharine Hurley, Whitney Isenhower, Enid Kaabunga, Baraka D Kaaya, Esther Kabui, Beth Kangwana, Lason Kapata, Henry Kaula, Gloria Kigo, Irene Kyomuhangi, Aliza Lailari, Sandra LeFevre, Megan Littrell, Greta Martin, Daniel Michael, Erik Monroe, Godefroid Mpanya, Felton Mpasela, Felix Mulama, Anne Musuva, Julius Ngigi, Edward Ngoma, Marjorie Norman, Bernard Nyauchi, Kathryn A. O’Connell, Carolyne Ochieng, Edna Ogada, Linda Ongwenyi, Ricki Orford, Saysana Phanalasy, Stephen Poyer, Justin Rahariniaina, Jacky Raharinjatovo, Lanto Razafindralambo, Solofo Razakamiadana, Christina Riley, John Rodgers, Andria Rusk, Tanya Shewchuk, Simon Sensalire, Julianna Smith, Phok Sochea, Tsione Solomon, Raymond Sudoi, Martine Esther Tassiba, Katherine Thanel, Rachel Thompson, Mitsuru Toda, Chinazo Ujuju, Marie-Alix Valensi, Vamsi Vasireddy, Cynthia B. Whitman, Cyprien Zinsou, Kara Hanson, Catherine Goodman

**Affiliations:** 1 Population Services International, 1120 19th St NW Suite 600, Washington, DC 20036 USA; 2 London School of Hygiene and Tropical Medicine, 15-17 Tavistock Place, London, WC1H 9SH UK

**Keywords:** Malaria test, Rapid diagnostic test, Microscopy, Price, Market share, Availability, sub-Saharan Africa

## Abstract

**Background:**

The World Health Organization guidelines have recommended that all cases of suspected malaria should receive a confirmatory test with microscopy or a malaria rapid diagnostic test (RDT), however evidence from sub-Saharan Africa (SSA) illustrates that only one-third of children under five with a recent fever received a test. The aim of this study was to evaluate availability, price and market share of microscopy and RDT from 2009/11 to 2014/15 in 8 SSA countries, to better understand barriers to improving access to malaria confirmatory testing in the public and private health sectors.

**Results:**

Repeated national cross-sectional quantitative surveys were conducted among a sample of outlets stocking anti-malarial medicines and/or diagnostics. In total, 169,655 outlets were screened. Availability of malaria blood testing among all screened public health facilities increased significantly between the first survey wave in 2009/11 and the most recent in 2014/15 in Benin (36.2, 85.4%, p < 0.001), Kenya (53.8, 93.0%, p < 0.001), mainland Tanzania (46.9, 89.9%, p < 0.001), Nigeria (28.5, 86.2%, p < 0.001), Katanga, the Democratic Republic of the Congo (DRC) (76.0, 88.2%, p < 0.05), and Uganda (38.9, 95.6%, p < 0.001). These findings were attributed to an increase in availability of RDTs. Diagnostic availability remained high in Kinshasa (the DRC) (87.6, 97.6%) and Zambia (87.9, 91.6%). Testing availability in public health facilities significantly decreased in Madagascar (88.1, 73.1%, p < 0.01). In the most recent survey round, the majority of malaria testing was performed in the public sector in Zambia (90.9%), Benin (90.3%), Madagascar (84.5%), Katanga (74.3%), mainland Tanzania (73.5%), Uganda (71.8%), Nigeria (68.4%), Kenya (53.2%) and Kinshasa (51.9%). In the anti-malarial stocking private sector, significant increases in availability of diagnostic tests among private for-profit facilities were observed between the first and final survey rounds in Kinshasa (82.1, 94.0%, p < 0.05), Nigeria (37.0, 66.0%, p < 0.05), Kenya (52.8, 74.3%, p < 0.001), mainland Tanzania (66.8, 93.5%, p < 0.01), Uganda (47.1, 70.1%, p < 0.001), and Madagascar (14.5, 45.0%, p < 0.01). Blood testing availability remained low over time among anti-malarial stocking private health facilities in Benin (33.1, 20.7%), and high over time in Zambia (94.4, 87.5%), with evidence of falls in availability in Katanga (72.7, 55.6%, p < 0.05). Availability among anti-malarial stocking pharmacies and drug stores—which are the most common source of anti-malarial medicines—was rare in all settings, and highest in Uganda in 2015 (21.5%). Median private sector price of RDT for a child was equal to the price of pre-packaged quality-assured artemisinin-based combination therapy (QAACT) treatment for a two-year old child in some countries, and 1.5–2.5 times higher in others. Median private sector QAACT price for an adult varied from having parity with an RDT for an adult to being up to 2 times more expensive. The exception was in both Kinshasa and Katanga, where the median price of QAACT was less expensive than RDTs.

**Conclusions:**

Significant strides have been made in the availability of testing, mainly through the widespread distribution of RDT, and especially in public health facilities. Significant barriers to universal coverage of diagnostic testing can be attributed to very low availability in the private sector, particularly among pharmacies and drug stores, which are responsible for most anti-malarial distribution. Where tests are available, price may serve as a barrier to uptake, particularly for young children. Several initiatives that have introduced RDT into the private sector can be modified and expanded as a means to close this gap in malaria testing availability and promote universal diagnosis.

**Electronic supplementary material:**

The online version of this article (doi:10.1186/s12936-017-1829-5) contains supplementary material, which is available to authorized users.

## Background

Since 2010, the World Health Organization (WHO) guidelines have recommended that all cases of suspected malaria should receive a confirmatory test with microscopy or a rapid diagnostic test (RDT) to confirm the diagnosis [1] and since 2012, this recommendation has been promoted through WHO’s ‘test, treat, and track’ strategy. Although malaria remains a public health concern, resulting in over 300,000 deaths in children under five [1], recent control efforts have significantly reduced the disease burden, especially in high transmission areas of Africa [2]. The decreased prevalence of malaria has emphasized the need to move away from presumptive treatment. This, together with the introduction of RDT technology, has allowed for malaria diagnosis to take place in remote and resource-poor settings [3], and has influenced the shift in policy to confirmatory testing [4].

Confirmatory testing of suspected cases of malaria with microscopy or RDT prior to treatment has the potential to enhance accurate diagnosis, and improve treatment of malaria and other febrile illnesses and reduce wastage of anti-malarial drugs [5]. Confirmatory testing has been shown to decrease the inappropriate use of the effective artemisinin-based combination therapy (ACT), which is the first-line treatment for the majority of countries in sub-Saharan Africa (SSA) [1]. It is thought that reducing inappropriate use of this treatment will impede the rate of resistance to ACT and decrease how much governments will need to spend on purchasing and supplying these medicines to health facilities [6–9].

National guidelines across SSA stipulate confirmatory testing prior to treatment, and national malaria control programmes and their partners have implemented strategies to improve access to testing through the scale up of RDT, particularly in the public sector [10]. Despite this, current evidence from population-based surveys suggests that confirmatory testing rates with microscopy or RDT remain low. According to recent household surveys conducted between 2013 and 2015 in SSA, the proportion of children under five with recent fever who received a malaria test was just 31% (Interquartile range [IQR] 19–40%) [1].

Current and comprehensive information about availability, price and the relative sale or distribution of RDT versus microscopy in the public and private sectors for diagnostic testing in SSA is needed. This will help to inform and monitor strategies to increase the proportion of suspected malaria cases receiving a confirmatory test. However, this information has to date not been available. Most studies that evaluate accessibility to malaria diagnostic testing tend to be small in scale, not nationally representative, focus on either the public or private sector only, or observe only one time point [11–15]. A more recent multi-country ACTwatch assessment of malaria diagnostic testing availability across the public and private sectors, published in 2015, focused solely on RDT availability in eight SSA countries during 2011 [16]. Other data on availability, price and market share of microscopy and RDT have been made available through multiple survey rounds, but data have not yet been collated across countries and presented overtime for key indicators.

ACTwatch was launched in 2008 by Population Services International (PSI), with support from the Bill and Melinda Gates Foundation, UNITAID and the UK Department for International Development (DFID) and was implemented in collaboration with the London School of Hygiene and Tropical Medicine. The goal of the project was to generate timely, relevant, and high quality evidence about anti-malarial markets for policy makers, donors, and implementing organizations. The aim of this paper is to address gaps in information concerning public and private sector availability, price and market share of microscopy and RDTs across eight malaria-endemic countries in SSA (Benin, the Democratic Republic of the Congo [DRC], Nigeria, Kenya, mainland Tanzania (subsequently referred to as Tanzania), Uganda, Madagascar and Zambia). Data collected by the ACTwatch project over multiple time points between 2009/11 and 2014/15 are presented to describe significant trends in availability, and to summarize private sector price and relative market share of RDT and microscopy for the public and private sector. Policy regarding private sector permission to use RDT varies by country and outlet type (see Table 1) and data are also presented to describe differences between private sector outlet types. The results will be useful to inform, monitor, and evaluate policies and strategies designed to improve access and use of malaria diagnostic testing.

**Table 1:** National policy regarding permission to administer RDT across private sector outlet types

	Private for-profit health facilities	Pharmacies and drug stores
West and Central Africa		
Benin	Only accredited private health facilities are permitted to administer testing	
DRC	Permitted to administer RDT	Pharmacies with a licensed pharmacist are permitted to administer RDT. Other drug stores are not allowed to administer RDT
Nigeria	Permitted to administer RDT	Drug stores, or Patient Propriety Medicines Vendors (PPMV) as they are called in Nigeria, were granted approval to administer RDT in 2015 at national level. Approval at sub-national level varies across states. Pharmacies are allowed to administer RDT within approved project pilots as of 2014
East Africa		
Kenya	Permitted to administer RDT	Not permitted to administer RDT. Pharmacies are allowed to administer RDT within approved project pilots as of 2014
Tanzania	Permitted to administer RDT	Accredited drug dispensing outlets (ADDOs) are allowed to administer RDT within approved project pilots. Policy granting permission to administer RDT for ADDOs is under review
Uganda	Permitted to administer RDT	Drug stores and pharmacies are allowed to administer RDT within approved project pilots. Policy granting permission to administer RDT is under review
Southern Africa		
Madagascar	Permitted to administer RDT	Pharmacies and drug stores permitted to administer RDT nationwide since 2014
Zambia	Permitted to administer RDT	Not permitted to administer RDT

## Methods

### Design and sampling

ACTwatch outlet surveys are nationally-representative (with the exception of the sub-national surveys in the DRC), cross-sectional quantitative surveys conducted among a sample of outlets stocking anti-malarial medicines and diagnostics. Detailed ACTwatch project and methodological information have been published elsewhere [17, 18].

All categories of outlets with the potential to stock anti-malarials in both the public and private sector were included in the study. Potential outlets include public and private outlets that may be likely to stock anti-malarial medicines or diagnostics according to each country context. In the public sector, this included government and non-government not-for-profit health facilities (hospitals, health centres, clinics, and health posts) and community health workers. Outlets sampled in the private sector included private for-profit health facilities (hospitals, health centres, and clinics), pharmacies, drug stores (registered/regulated and unregistered/unregulated), general retailers selling fast-moving consumer goods and itinerant drug vendors (mobile vendors without a fixed service delivery point).

Lists of all potentially eligible outlets were not routinely available and therefore a cluster sampling approach with an outlet census was used to identify outlets for inclusion. Clusters were administrative units ideally with a size of 10,000–15,000 inhabitants, and were selected using probability proportional to population size sampling. Within each selected cluster all outlets with the potential to provide anti-malarials to consumers were screened for eligibility. Outlets were eligible for an anti-malarial product audit if they had one or more anti-malarials in stock on the day of the survey or/and malaria diagnostic testing.

Boundaries for the outlet census were typically extended to higher administrative units to cover a larger area for the census of public health facilities and pharmacies, in order to over-sample these relatively uncommon but important outlet types.

Each survey was stratified to deliver estimates for relevant research domains: all countries had urban and rural stratification, with the exception of Nigeria for which six geopolitical zones were used as research domains. Each study round was powered to detect a minimum of a 20-percentage point change in availability of quality-assured ACT (QAACT) among anti-malarial stocking outlets between each round and between domains in a given round at the 5% significance level with 80% power. The number of study clusters was calculated for each research domain based on the required number of anti-malarial stocking outlets and assumptions about the number of anti-malarial stocking outlets per cluster. Sample size requirements for follow-up surveys were calculated using information from previous survey rounds including anti-malarial and QAACT availability, outlet density per cluster, and design effect.

Data collection periods varied by country and over time but were typically implemented during the peak malaria transmission season for each country and lasted between 6 weeks and 2 months. Efforts were made to ensure surveys were implemented over similar time points across the survey rounds.

### Training and fieldwork

Interviewer training consisted of standardized classroom presentations and exercises as well as a field exercise. Exams administered during training were used to select data collectors, supervisors, and quality-controllers. Additional training was provided for supervisors and quality-controllers and focused on field monitoring, verification visits, and census procedures. Data collection teams were provided with a list of selected clusters and official maps that illustrated their administrative boundaries. In each selected cluster, data collectors conducted a full enumeration of all outlets that had the potential to provide anti-malarials and/or malaria blood testing. This included enumeration of outlets with a physical location, as well as identification of community health workers and itinerant drug vendors using local informants and snowball sampling. The primary provider/owner of each outlet was invited to participate in the study, and screening questions were administered to assess anti-malarial or/and malaria diagnostic availability. Interviews were conducted in local language using questionnaires that were translated from English to the local language using a forwards and backwards blind translation. Quality control measures implemented during data collection included questionnaire review by supervisors, and spot checks by quality controllers on 10–20% of all outlets.

### Measures

The outlet survey questionnaire included an audit of all available RDT. Providers were asked to show the interviewer all RDT that were available in the outlet. A product audit sheet captured information for each unique RDT in the outlet, including brand name, manufacturer, and country of manufacture. Providers were asked to report the retail and wholesale price for each RDT as well as the number of RDT distributed/administered to individual customers in the previous week. Providers additionally reported on malaria microscopy services including availability, price, and number of individuals tested for malaria by microscopy in the previous week (see Additional file 1 for a sample of the ACTwatch questionnaire). All surveys were paper-based with the exception of Madagascar (2015) and Uganda (2015), where data were collected using Android phones and forms created using DroidDB (© SYWARE, Inc., Cambridge, MA, USA).

### Protection of human subjects

The outlet survey protocols received ethical approval from national ethical approval boards within each country. Provider interviews and product audits were completed only after administration of a standard informed consent form and provider consent to participate in the study. Providers had the option to end the interview at any point during the study. Standard measures were employed to maintain provider confidentiality and anonymity.

### Data analysis

Double data entry was conducted using Microsoft Access (Microsoft Corporation, Redmond, WA, USA) with built-in range and consistency checks. Data were analyzed across survey rounds using Stata (StataCorp College Station, TX).

Standard indicators were constructed according to definitions applied across the ACTwatch project which have been described elsewhere [17, 18]. Availability of RDT was defined as presence of one or more RDT at the outlet at the time of the survey. Availability of microscopy was defined as provider report of availability of malaria microscopy testing services. Functionality of the microscope and availability of slides and Giemsa stain were not confirmed. Availability of malaria testing was calculated among all screened outlets for public health facilities, and among anti-malarial stockists for all other outlet types. An anti-malarial stockist was defined as an outlet with one or more anti-malarials in stock on the day of the survey, or reportedly in stock within the past 3 months. Significant differences in test availability levels between years in each country were estimated using logistic regression, with a binary dependent variable for availability of testing at the outlet level, and a dummy independent variable for year.

The private sector price of a malaria test using microscopy or RDT was assessed through provider reports of consumer prices for the last survey round. Price data presented were collected in local currencies and converted to United States dollars using official exchange rates for the period of data collection. Providers were asked to report the total cost of testing to a customer including any consultation or service fees. Median private sector price for RDTs was calculated and reported with the IQR as a measure of dispersion. Median estimates were not reported on in cases of low sample size, specifically where the N contributing to a median price estimate was below 20. Price of testing was compared with median price for pre-packaged treatment with a first-line QAACT. In countries with two first-line ACT, the most common was selected for the median price comparison. The most common pre-packaged QAACT treatment for an adult was artemether-lumefantrine (AL) in all countries except Madagascar and Kinshasa (DRC) and Katanga (DRC) where it was artesunate–amodiaquine (ASAQ). The price of testing for an adult was compared to the price of pre-packaged therapy for a 60 kg adult (e.g. AL 20/120 package size of 24 tablets). The price of testing for a child under five was compared to the price of pre-packaged therapy for a 10 kg child (e.g. AL 20/120 package size of 6 tablets).

QAACT were ACT products meeting one of three criteria: (1) the product had WHO pre-qualified status; (2) the product was in compliance with the Global Fund quality assurance policy and appeared on the Global Fund list of approved products for procurement; or (3) the product was granted regulatory approval by the European Medicines Agency.

Provider reports on the number of people tested using RDT or malaria microscopy during the week preceding the survey were used to calculate the relative market share for the public and private sector and for the type of test (RDT and microscopy). The relative market share for a sector or test type is the number of tests that were reportedly performed on each customer during the week preceding the survey for the sector/test type as a percentage of all tests performed across sectors.

Outlets were grouped into three main categories: (1) public health facilities consisting of government and private not-for-profit facilities; (2) private for-profit health facilities; and (3) pharmacies and drug stores. Results are presented separately for private for-profit health facilities and pharmacies/drug stores given the differences in testing policy for these outlet types (Table 1). Additional file 2 includes findings for other outlet types including community health workers, general retailers, and itinerant drug vendors, given the general low availability of malaria blood testing among these outlet types.

Sampling weights were calculated as the inverse of the probability of cluster selection. All point estimates were weighted using survey settings and all standard errors calculated taking account of the clustered and stratified sampling strategy with Stata survey commands.

## Results

A total of 198,836 outlets were screened to assess availability of anti-malarials and blood testing across the eight countries and 28 survey rounds between 2009/11 and 2014/15. Anti-malarials were available on the day of the survey or within the previous 3 months among a total of 52,312 outlets. In total, 11,981 RDT were audited (Table 2).

**Table 2:** Results of the outlet census and RDT audit by country and survey year

Country	Year	Screened (N of outlets)	Anti-malarial stockist^a^ (N of outlets)	RDT products audited^b^ (N of products)
West and Central Africa				
Benin	2011	2891	1413	96
	2014	4332	1939	239
Kinshasa (DRC)	2009	2368	777	n/a
	2013	3364	977	79
	2015	1168	1078	267
Katanga (DRC)	2013	2270	785	140
	2015	1052	1027	435
Nigeria	2009	5456	2160	n/a
	2011	7938	1548	44
	2013	5148	1784	448
	2015	13,480	3568	489
East Africa				
Kenya	2010	13,897	2554	75
	2011	11,383	2084	144
	2014	12,676	2405	654
Tanzania	2010	3120	650	28
	2011	3702	798	41
	2014	4724	2138	504
Uganda	2010	11,153	2499	180
	2011	16,207	3226	843
	2013	7932	3472	1573
	2015	9438	4598	2267
Southern Africa				
Madagascar	2010	6769	2593	n/a
	2011	10,046	2790	722
	2013	10,149	1906	1087
	2015	13,481	1203	699
Zambia	2009	3378	459	n/a
	2011	5436	860	278
	2014	5878	1021	649

*n/a* not applicable, indicates years during which RDT availability was assessed but an audit of all available RDT was not conducted

^a^Outlets with at least one anti-malarial in stock on the day of the survey or within the past 3 months (completed interview)

^b^Represents the number of RDT products that were audited at an outlet during each survey round

### Malaria blood testing availability

**Fig. 1:**
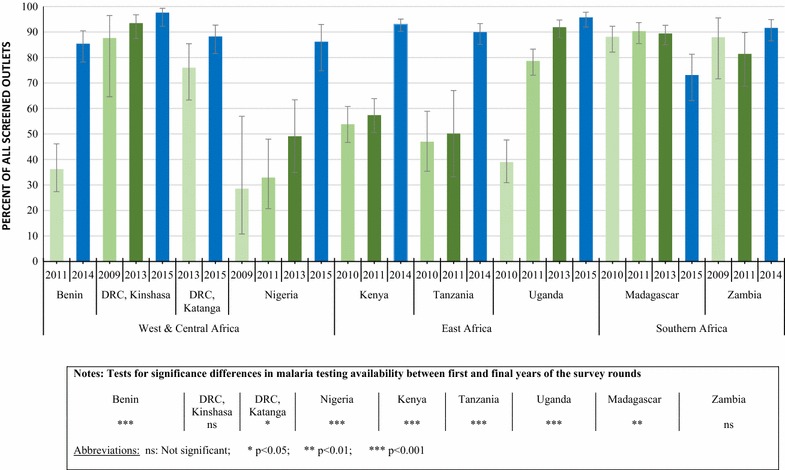
Availability of malaria blood testing among all screened public health facilities (Public health facilities are inclusive of government and non-government not-for-profit hospitals, clinics, health centers and health posts), 2009/11–2014/15

The availability of malaria blood testing (RDT or microscopy) among all screened public health facilities increased significantly between 2009/11 and 2014/15 in Benin (36.2, 85.4%, p < 0.001), Kenya (53.8, 93.0%, p < 0.001), Tanzania (46.9, 89.9%, p < 0.001), Nigeria (28.5, 86.2%, p < 0.001), Katanga (76.0, 88.2%, p < 0.05), and Uganda (38.9, 95.6%, p < 0.001). Testing availability was not significantly different between survey rounds in Kinshasa (87.6, 97.6%) and Zambia (87.9, 91.6%). Testing availability significantly decreased in Madagascar (88.1, 73.1%, p < 0.01) (Fig. 1).

A significant increase in availability of RDT was found in Benin (30.5, 75.4%, p < 0.001), Kenya (8.7, 62.6%, p < 0.001), Tanzania (20.8, 76.2%, p < 0.001), Kinshasa (2.9, 50.4%, p < 0.001), Katanga (47.8, 84.7%, p < 0.001), Nigeria (8.6, 83.4%, p < 0.001), and Uganda (4.0, 83.6%, p < 0.001) (Table 3). In Madagascar there was a significant decline in testing availability of RDT (Table 3).

**Table 3:** Percentage of all screened public health facilities^a^ with RDT and microscopy available on the day of the survey, over time

	n	% Outlets stocking RDT (95% CI)	% Outlets with microscopy available (95% CI)
West and Central Africa			
Benin			
2011	257	30.5 (21.3, 41.7)	8.8 (5.0, 15.1)
2014	263	75.4 (64.6, 83.8)***	17.8 (11.4, 26.8)*
Kinshasa (DRC)			
2009	27	2.9 (0.5, 14.2)	86.2 (66.1, 95.3)
2013	89	23.5 (13.3, 38.1)	89.3 (81.0, 94.3)
2015	277	50.4 (39.9, 61.0)***	91.2 (84.5, 95.1)
Katanga (DRC)			
2013	97	47.8 (34.0, 61.9)	47.6 (36.3, 59.1)
2015	284	84.7 (77.2, 90.0)***	30.8 (24.2, 38,2)*
Nigeria			
2009	249	8.6 (1.8, 32.9)	28.5 (10.7, 56.9)
2011	109	14.9 (7.5, 27.7)	24.7 (14.8, 38.4)
2013	711	43.0 (29.6, 57.6)	12.6 (5.8, 25.4)
2015	210	83.4 (72.7, 91.0)***	27.8 (15.9, 44.2)***
East Africa			
Kenya			
2010	443	8.7 (4.6, 16.0)	49.8 (43.2, 56.4)
2011	474	18.5 (9.7, 32.4)	44.6 (36.1, 53.5)
2014	528	62.6 (55.6, 69.0)***	66.4 (62.2, 70.4)***
Tanzania			
2010	87	20.8 (10.1, 38.0)	28.1 (20.0, 38.0)
2011	64	38.6 (23.5, 56.3)	25.0 (15.4, 37.8)
2014	336	76.2 (69.4, 81.9)***	32.3 (26.2, 39.0)
Uganda			
2010	811	4.0 (1.7, 9.2)	36.2 (28.8, 44.3)
2011	718	51.2 (44.7, 57.6)	47.8 (41.5, 54.2)
2013	728	79.5 (71.8, 85.5)	41.4 (36.1, 47.0)
2015	334	83.6 (75.4, 89.5)***	59.0 (51.1, 66.6)***
Southern Africa			
Madagascar			
2010	524	87.4 (81.3, 91.6)	3.7 (2.1, 6.3)
2011	669	89.7 (84.9, 93.1)	5.7 (4.6, 7.2)
2013	620	87.4 (82.7, 91.0)	14.7 (7.7, 26.2)
2015	273	72.0 (62.0, 80.3)**	3.6 (2.3, 5.5)
Zambia			
2009	178	85.7 (70.4, 93.8)	40.6 (30.5, 51.6)
2011	294	68.4 (55.4, 79.0)	35.8 (24.9, 48.5)
2014	498	89.4 (83.8, 93.2)	22.8 (14.3, 34.3)*

^a^Inclusive of government and non-government not-for-profit hospitals, clinics, health centers and health posts

*CI* Confidence Interval

* p < 0.05; ** p < 0.01; *** p < 0.001, in reference to baseline year

**Fig. 2:**
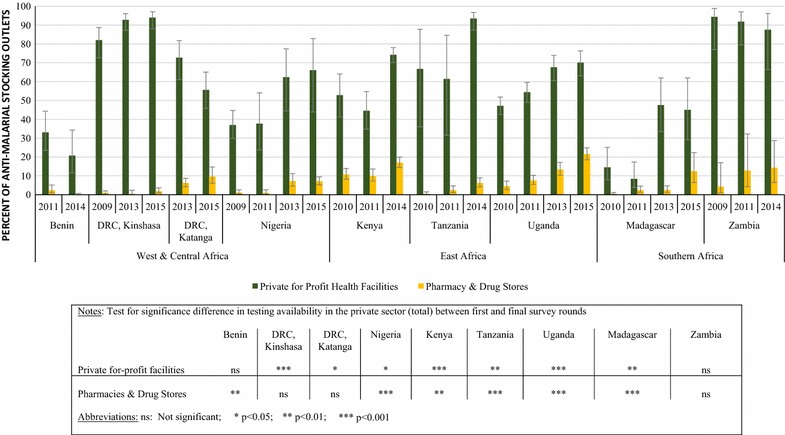
Availability of malaria blood testing among the anti-malarial stocking private sector, 2009/11–2014/15

In the anti-malarial stocking private sector, the availability of malaria blood testing (RDT or microscopy) among the private for-profit sector increased significantly between 2009/11 and 2014/15 in Kinshasa (82.1, 94.0%, p < 0.05), Nigeria (37.0, 66.0%, p < 0.05), Kenya (52.8, 74.3%, p < 0.001), Tanzania (66.8, 93.5%, p < 0.01), Uganda (47.1, 70.1%, p < 0.001) and Madagascar (14.5, 45.0%, p < 0.01). Blood testing availability did not change significantly over time in Benin (33.1, 20.7%). and Zambia (94.4, 87.5%). Significant declines were observed in Katanga (72.7, 55.6%, p < 0.05) (Fig. 2).

In comparison with private for-profit facilities, the availability of malaria blood testing among anti-malarial stocking pharmacies and drug stores was lower across all countries. During the most recent survey round, testing availability among anti-malarial stocking pharmacies and drug stores ranged from 0.1% in Benin to 21.5% in Uganda. Among pharmacies and drug stores, a significant increase in availability was observed between 2009/11 and 2014/15 in Nigeria (1.1, 7.1%, p < 0.001), Kenya (10.7, 17.0%, p < 0.05), Tanzania (0.4, 6.2%, p < 0.001), Uganda (4.5, 21.5%, p < 0.001), and Madagascar (0.4, 12.4%, p < 0.001) (Fig. 2).

Among private for-profit facilities, a significant increase in availability of RDT was observed in Nigeria (11.0, 42.4%, p < 0.05), Kenya (6.7, 29.3% p < 0.001), Uganda (9.7, 47.7%, p < 0.001), and Madagascar (12.5, 43.8%, p < 0.01). In Kenya, significant increases in microscopy availability were observed (48.5, 61.1%, p < 0.05). In Katanga, malaria microscopy availability showed significant declines (49.2, 23.3%, p < 0.01) (Table 4). Among pharmacies and drugs stores, a significant increase in availability of RDT was observed in Nigeria (0.5, 7.0%, p < 0.05), Kenya (2.2, 12.8% p < 0.001), Tanzania (0.4, 5.7%, p < 0.01), Uganda (2.2, 20.7%, p < 0.001), and Madagascar (0.45, 12.4%, p < 0.001) (Table 4). Microscopy was rarely available among pharmacies and drug stores in all countries with the exception of Kenya (6.1% in 2014) (Table 4).

**Table 4:** Percentage of anti-malarial-stocking private sector outlets with malaria RDT and microscopy available on the day of the survey over time

	Private for-profit health facilities^a^			Pharmacies and drug stores		
	N	% Outlets stocking RDT (95% CI)	% Outlets with microscopy (95% CI)	N	% Outlets stocking RDT (95% CI)	% Outlets with microscopy (95% CI)
West and Central Africa						
Benin						
2011	134	11.2 (2.4, 38.9)	22.5 (14.4, 33.4)	221	2.2 (9.8, 5.1)	0.0
2014	139	11.6 (5.2, 23.8)	10.5 (4.0, 24.6)	192	0.1 (< 0.1, 0.4)***	0.1 (< 0.1, 0.4)
Kinshasa (DRC)						
2009	71	5.1 (1.9, 13.0)	81.5 (72.0, 88.2)	661	0.1 (0.0, 1.0)	0.9 (0.4, 2.0)
2013	200	18.3 (13.2, 25.0)	89.3 (84.2, 92.8)	693	0.4 (0.1, 2.3)	0.0
2015	176	31.1 (21.8, 42.2)	88.7 (83.2 92.5)	612	1.3 (0.5, 3.2)	0.1 (< 0.1, 1.9)
Katanga (DRC)						
2013	134	45.4 (33.4, 58.0)	49.2 (35.2, 63.3)	529	5.4 (3.7, 7.7)	2.2 (1.0, 4.8)
2015	141	45.9 (36.5, 55.5)	23.3 (15.6, 33,4)**	565	9.4 (5.9, 14.6)	0.8 (0.4, 1.8)
Nigeria						
2009	367	11.0 (2.7, 36.0)	36.1 (29.4, 43.4)	1360	0.5 (0.1, 1.9)	0.6 (0.2, 1.8)
2011	93	8.6 (3.3, 20.6)	33.2 (19.5, 50.4)	1206	0.9 (0.3, 2.6)	0.2 (< 0.1, 0.9)
2013	78	46.9 (30.3, 64.2)	24.0 (12.0, 42.2)	885	6.9 (4.3, 11.0)	0.2 (0.1, 1.1)
2015	240	42.4 (29.3, 56.7)*	41.9 (18.3, 70.0)	2956	7.0 (5.2, 9.4) ***	<0.1 (< 0.1, 0.3)*
East Africa						
Kenya						
2010	269	6.7 (3.2, 13.6)	48.5 (37.7, 59.4)	655	2.2 (0.8, 6.0)	8.8 (5.8, 13.0)
2011	280	6.7 (3.7, 11.8)	40.2 (30.4, 50.8)	744	3.1 (1.8, 5.2)	7.6 (5.1, 11.2)
2014	375	29.3 (25.7, 33.0)***	61.1 (56.7, 65.4)*	1045	12.8 (11.0, 14.9)**	6.1 (4.8, 7.8)
Tanzania						
2010	10	8.8 (1.0, 49.1)	58.0 (27.0, 83.8)	455	0.4 (0.1, 1.5)	<0.1 (< 0.1, 0.2)
2011	25	3.4 (0.6, 17.8)	61.4 (31.6, 84.6)	673	1.0 (0.3, 3.3)	1.4 (0.7, 3.1)
2014	172	47.7 (40.1, 55.5)	83.1 (75.6, 88.6)	1599	5.7 (3.9, 8.1)***	0.7 (0.3, 1.8)**
Uganda						
2010	394	9.7 (6.6, 14.0)	41.3 (36.3, 46.6)	1220	2.2 (1.2, 3.8)	2.6 (1.2, 4.8)
2011	811	20.3 (15.7, 25.8)	43.5 (39.2, 47.8)	1544	5.1 (3.6, 7.2)	2.5 (1.4, 4.6)
2013	394	31.4 (25.9, 37.4)	46.6 (37.6, 55.8)	1512	11.2 (8.4, 14.8)	2.4 (1.4, 4.1)
2015	966	47.7 (41.7, 53.8)***	41.1 (34.3, 48.1)	2381	20.7 (17.8, 23.9)***	1.1 (0.6, 2.3)
Southern Africa						
Madagascar						
2010	71	12.5 (6.5, 22.9)	4.3 (2.1, 8.5)	324	0.4 (0.1, 1.1)	0.1 (< 0.1, 0.3)
2011	47	7.7 (3.5, 15.9)	3.6 (1.5, 8.4)	461	2.4 (1.2, 4.6)	0.0
2013	94	45.9 (32.9, 59.5)	9.7 (4.4, 20.4)	529	1.7 (1.0, 3.0)	0.7 (0.1, 5.0)
2015	182	43.8 (28.0, 60.9)**	3.1 (1.6, 5.7)	271	12.4 (6.5, 22.3)***	0.0
Zambia						
2009	33	78.3 (58.0, 90.5)	74.4 (57.8, 86.1)	189	4.2 (0.9, 17.1)	3.1 (0.4, 20.2)
2011	49	43.8 (23.6, 66.2)	86.9 (73.1, 94.2)	362	12.7 (4.3, 32.2)	0.1 (< 0.1, 0.6)
2014	22	68.1 (45.6, 84.4)	72.2 (42.0, 90.3)	354	14.3 (6.5, 28.7)	0.4 (0.1, 0.9)

^a^Private for-profit health facilities (hospitals, clinics and health centres) and pharmacies or drug stores with anti-malarials in stock on the day of the survey or within the previous 3 months

* p < 0.05 ** p < 0.01 *** p < 0.001, in reference to baseline year

In some study countries, community health workers, general retailers, and itinerant drug vendors stocking anti-malarials were also stocking RDT. Among anti-malarial stocking community health workers, RDT availability was 58.2% during the most recent survey round in Uganda, 72.8% in Madagascar and 83.2% in Zambia. Availability among general retailers and itinerant drug vendors was generally very low (<5%) with the exception of 8.5% observed during the most recent survey in Tanzania (Additional file 2).

### Private sector price of malaria blood testing relative to treatment

The median private sector price of the most common pre-packaged QAACT treatment for an adult was 2 times more expensive than the median price of adult RDT testing in Tanzania, Uganda, and Zambia, and 1.25 more expensive in Nigeria. QAACT and RDT testing were the same price in Kenya and Madagascar. In both Kinshasa and Katanga, the median price of QAACT ($0.00 and $0.55, respective) was less expensive than RDT testing (both $1.10). The low price of adult QAACT in Kinshasa is attributed to low availability of products (N = 40), which were part of small scale subsidy initiatives. 

**Fig. 3:**
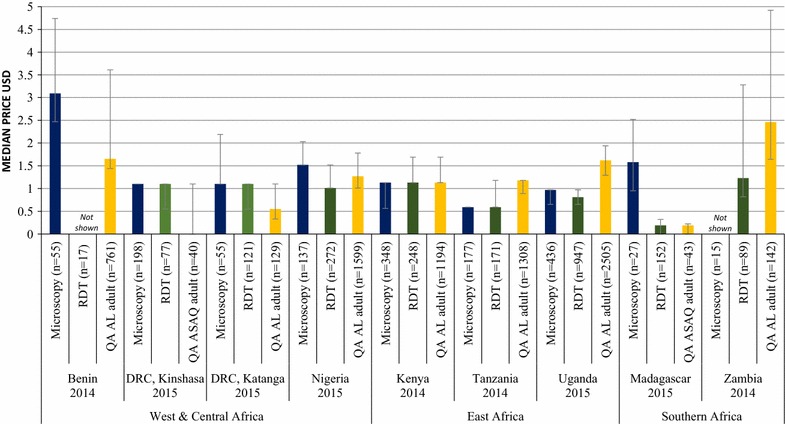
Median private sector price for malaria testing for an adult and quality-assured adult ACT treatment

Malaria microscopy was more expensive than QAACT for adults in Benin (1.9 times more expensive), Katanga (2 times more expensive), and Kinshasa (where median price of treatment was $0.00), Nigeria (1.2 times more expensive), and Madagascar (8.3 times more expensive), and was the same price as treatment in Kenya. QAACT was more expensive than microscopy in Tanzania (2 times more expensive) and Uganda (1.7 times more expensive) (Fig. 3).

**Fig. 4:**
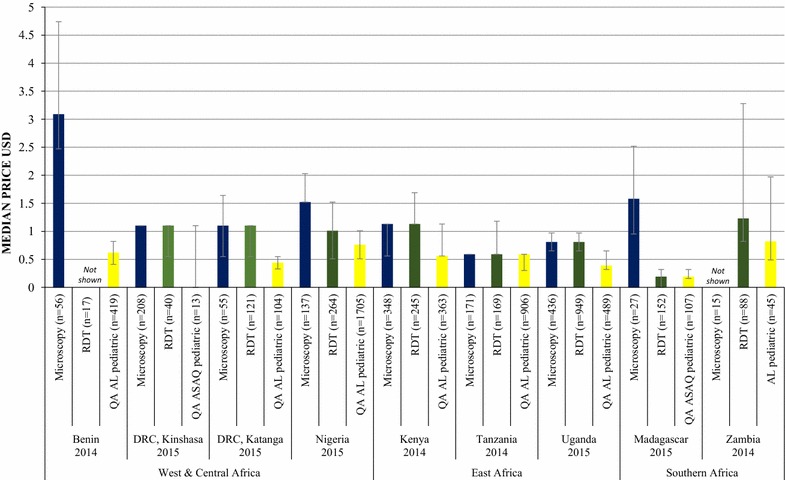
Median private sector price for malaria testing for a child and quality-assured pediatric ACT treatment

The median private sector price of RDT testing for a child was higher than the price of pre-packaged QAACT treatment for a 2-year old child in Katanga (2.5 times more expensive), Nigeria (1.4 times more expensive), Kenya (2 times more expensive), Uganda (2.1 times more expensive), and Zambia (1.5 times more expensive). The prices of treatment and of RDT for a child were the same in both Tanzania and Madagascar. Malaria microscopy for a child was also more expensive than pre-packaged pediatric treatment in Benin (4.9 times more expensive), Katanga (3.3 times more expensive), Nigeria (4.2 times more expensive), Kenya (2 times more expensive), Uganda (2.1 times more expensive), and Madagascar (8.3 times more expensive). The prices of QAACT and of microscopy for a child were the same in Tanzania (Fig. 4). In Kinshasa, the median price of prepackaged QAACT for a 2-year-old child was $0.00, meaning that median price for both RDT and microscopy was higher than for treatment here. The low price of QAACT for a 2-year-old child in Kinshasa is attributed to low availability of products (N = 13), which were part of small scale subsidy initiatives.

Private sector prices for quality-assured pre-packaged ACT treatment and testing disaggregated by outlet type (private facility, pharmacy/drug store) are provided in Additional file 3.

### Malaria blood testing market share

**Fig. 5:**
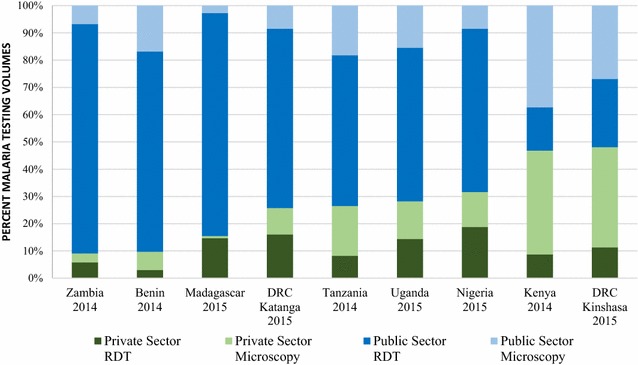
Malaria testing market share, by test type and across sectors

At the time of the most recent survey round, the majority of malaria testing was performed in the public sector in Zambia (90.9%), Benin (90.3%), Madagascar (84.5%), Tanzania (73.5%), Uganda (71.8%), Nigeria (68.4%), Kenya (53.2%), and the DRC, where the public sector was responsible for 74.3% of tests performed in Katanga and 51.9% in Kinshasa. The majority of malaria blood testing was performed with RDT in Zambia (89.9%), Benin (76.5%), Madagascar (96.5%), Tanzania (63.5%), Uganda (70.8%), Nigeria (78.7%), and Katanga (81.9%). Microscopy performed by both the public and private sectors accounted for the majority of malaria testing in Kenya (75.4% inclusive of 37.3% public and 38.1% private) and Kinshasa (63.7% inclusive of 26.9% public and 36.8% private) (Fig. 5).

## Discussion

The results from this study show that significant strides have been made in improving availability of malaria testing, especially in public health facilities, where more than 80% of facilities stocked any test across most countries during the last survey round. However, population based evidence suggests that testing uptake remains low, with only about one-third of children under five with recent fever receiving a malaria test [19]. The findings from this survey suggest that this may be attributed to a lack of private sector availability of testing and price barriers, particularly in pharmacies and drugs stores, where most patients seek treatment in SSA.

### Limitations

The main strength of this paper is that data are presented at a national level, with the exception of the DRC where only Katanga and Kinshasa provinces were surveyed partially due to poor infrastructure that limited travel, and to insecurity in other regions of the country. In addition, this study assessed availability, price, and market share in both the public and private sector, providing a complete picture of the malaria testing market. However, several limitations exist. Information on pricing was obtained by asking the provider how much they would charge for a test, which may have exposed the data to respondent bias as providers may state a price they consider more favorable to the interviewers. Another limitation is that the quality of the RDT or functionality of microscopic tests stocked was not assessed, nor was expiry information on stocked RDT collected. In addition, while tests may have been available at an outlet, the length of waiting time for the results was not assessed, though one would expect this to be longer for microscopy than for RDTs. Other limitations specific to the ACTwatch methodology are described in more detail elsewhere [20].

### Public health facilities

Increases in testing availability were observed among public health facilities in several countries. At least nine in ten public sector facilities had malaria testing available in Kinshasa (DRC), Kenya, Tanzania, Uganda, and Zambia, and availability was greater than 85% in Benin and Katanga (DRC). Large increases in availability over time were observed across most countries, and this was most striking in Benin and Tanzania between the last two survey rounds, where availability increased by almost fifty percentage points. Statistically significant differences were found between the first and final surveys for all country contexts, with the exception of Kinshasa and Zambia. These increases were largely achieved through the extensive deployment of RDT. Market share data, presented for the last survey round, also illustrated that the majority of confirmatory tests (up to 90%) were being carried out in public health facilities, exemplifying the importance of this sector as a source of confirmatory testing. In addition, across country contexts, the most common type of test carried out in public health facilities was RDT, with some exceptions noted in Kenya and Kinshasa where microscopy was more commonly administered and available.

These findings reflect policies of confirmatory diagnosis that many African countries have adopted since WHO changed its malaria treatment policy to ‘test, treat, and track.’ Several countries have implemented programmes aimed at expanding access to and use of diagnostic testing among public health facilities, largely focusing on RDT given evidence that these can be effectively performed even at lower levels of the health system [21]. The findings presented here are commensurate with public sector surveillance data from the region, indicating that the proportion of suspected malaria cases receiving a confirmatory test in the public sector increased from 40% in 2010 to 76% in 2015 [1]. Again this is attributed to an increase in the use of RDT, which accounted for 74% of diagnostic testing among suspected cases in 2015 [1]. This trend of increased confirmatory testing among public health facilities is also evident in the results of household surveys, where the proportion of febrile children who received a malaria diagnostic test in the public sector rose from a median of 29% in 2010 (IQR: 19–46%) to a median of 51% in 2015 (IQR: 35–60%) [1]. Similarly, distribution of RDT from national malaria control programmes in SSA to supply the public sector has also increased over the years, and a total of 179 million were distributed to the public sector in 2015 [1].

Despite these improvements over time among public health facilities, the findings from the last survey round demonstrate that gaps persist and 100% coverage of confirmatory diagnosis has not yet been achieved. In Madagascar, only three-quarters of public health facilities had testing available, reflecting a decline in recent years and a significant reduction since the baseline survey. Stock-outs are known to be a common problem facing the public health sector, and this has been demonstrated with inconsistent supplies of ACT [22]. In Madagascar, the reduction in availability of RDT among public health facilities may be explained by a delay in funding, with orders of RDTs only arriving in country in July 2015 after several months of stock-outs. Indeed, several challenges have been reported with maintaining constant public sector supply including the lack of technical capacity, archaic procurement methods, and cumbersome tendering processes [23]. Investments to strengthen both the supply system and the health information system, using systems such as the District Health Information Software (DHIS2), will assist in tracking RDT availability and lend to a more streamlined, demand driven, and accountable procurement and supply chain system [24].

### Private sector

In contrast to the public sector, there has been very little progress in testing scale up in the anti-malarial stocking private sector, particularly among pharmacies and drug stores—which are the most common source of anti-malarial medicines [18]. The percentage of outlets stocking any test in pharmacies and drug stores remained negligible across most country contexts, and was highest in Uganda in 2015 where 21% of pharmacies/drugs stores had a test in stock. Availability in the private sector was somewhat higher among private for-profit facilities, where modest improvements in the percentage of outlets stocking any test were observed, driven by a rise in RDT distribution. The findings from the outlet survey mirror evidence from population-based surveys in SSA that illustrate how the proportion of febrile children who received a malaria diagnostic test was greater if they sought care in the public sector (median: 51%, IQR: 35–60%) than in the formal private sector (median: 40%, IQR: 28–57%) or in the informal private sector (median: 9%, IQR: 4–12%) [1].

The low availability of malaria tests in pharmacies and drug stores is a key barrier to improving universal access to confirmatory testing since these outlet types are an important source of malaria treatment. For example, in Nigeria, over 70% of anti-malarial medicines were distributed through drug stores [25], known as PPMVs, yet less than 10% of these outlets had malaria testing available. Similarly in Tanzania, almost half of the total market share was distributed by pharmacies and drug stores (ADDOs), but only 6% had confirmatory testing available [26].

Low private sector availability of malaria testing can partly be explained by national regulatory frameworks, which restrict testing in these outlet types. However, there is a growing body of evidence that malaria case management can be well administered among certain outlet types in the private sector. In Tanzania, a randomized controlled trial to investigate whether the introduction of RDT among ADDOs improved malaria case management found that confirmatory diagnosis increased from 19 to 74% in intervention districts, which also resulted in improved targeting of ACT to patients with malaria [12]. Similar positive outcomes have been demonstrated among Licensed Chemical sellers in Ghana (private retail sector shops) [27] and drug shops in Uganda [28]. As such, several strategies have been piloted in SSA countries to facilitate access to confirmatory testing within pharmacies and drug stores. It has been shown that these outlets can safely and correctly test for malaria with appropriate training, supervision, and record keeping [29]. This suggests that the policy in favor of confirmatory testing in pharmacies and drug stores may foster increased access and appropriate case management of suspected malaria cases.

However, scaling up malaria testing within the private sector is not without challenges. While national scale implementation of RDT in the private sector has not yet been observed in SSA, this has been implemented in Cambodia over the past 10 years. A review of Cambodia’s private sector strategy has pointed to several challenges in maintaining constant supply of RDT and determining effective incentives for private providers and patients to use RDTs and adhere to results [30]. Furthermore, scale-up of RDTs in the private sector is not without major logistical challenges to ensure appropriate provision and supply of these commodities and at the same time guarantee safe blood practices and appropriate disposal of RDT. There may also be inadvertent effects on the use of antibiotics. Studies from Zanzibar [31] and mainland Tanzania [32] have shown increased prescription rates for antibiotics when RDTs were introduced, particularly for negative cases. Scale-up of RDTs in the private sector would also require time and substantial financial resources, which some may argue could be better spent on supervising the public sector. Careful consideration of future private sector strategies is needed and approaches should be reviewed according to each country’s context and regulatory framework.

Prices were only assessed for the last survey round, and only in the private sector, since tests within the public sector should be free. In the private sector, the median private sector price of pre-packaged QAACT treatment for an adult was either the same price or up to two times more expensive than the median price of an RDT, with the exception of Kinshasa and Katanga where the median price of treatment was less expensive than RDT testing. In contrast, the median private sector price of RDT testing for a child was higher than or equal to the price of pre-packaged QAACT treatment for a 2-year old child across countries. The median microscopy prices were generally higher than the price of carrying out an RDT in both adults and children.

These findings illustrate that where testing is available in the private sector, there appears to be a financial incentive in many cases for adults to test before treatment. However, among children, the low cost of pre-packaged ACT relative to RDT means that there is less of a financial incentive to test before purchasing ACT for this age group.

It has been suggested that in order to improve uptake of testing and therefore targeted treatment, the cost of testing should be lower than the cost of ACT [9, 33]. One possible way of achieving this is to subsidize RDT in the private sector along with ACT. Previous empirical research has provided some support for a combined subsidy. For example, a study in Uganda showed that introducing subsidized RDT in drug shops, alongside training and community awareness programmes, was able to significantly improve appropriate treatment of malaria over time. RDT-positive patients were 23 percentage points more likely to buy ACT (p = .005) and 33.1 percentage points more likely to buy other antimalarials (p < .001) than RDT-negative patients[9]. Lessons learned from pilot studies have shown that such subsidies do increase uptake of RDT [33, 34], and are most effective when prices are at a level that will still create sufficient profit to encourage providers to offer testing as a service, and manufacturers to continue producing these products [9, 34, 35]. RDT subsidies will need to be supported with behavior change communication to safeguard the proper use of the tests by providers [34, 35] and to ensure febrile patients are encouraged to test prior to treatment.

Achieving high levels of confirmatory testing prior to treatment and ensuring rational ACT use will require solutions that include the private sector. As previously discussed, this may include the introduction of subsidized RDT into certain outlet types in the private sector such as drug stores that play a role in malaria case management. Indeed, several studies have provided evidence that acceptance of malaria testing is generally high, with most patients welcoming the idea of receiving treatment based on a confirmed diagnosis [36–38]. However, recent evidence has pointed to several challenges around the poor communication practices between providers and patients, and the testing process, including limited inter-personal exchange between providers and patients which can lead to poor malaria case management [39]. To overcome this, future strategies may benefit from clear provider protocols to enable a more effective patient assessment and discussion on test outcomes, to include reasons for carrying out the test, particularly among RDT-negative patients [38]. There are also inherent challenges when patients test negative for malaria, particularly in the private sector, where providers may not have the qualifications or experience to know how to correctly manage the patient [30]. There is also evidence that providers do not always comply with testing guidelines and may treat patients with anti-malarials despite negative malaria tests for several reasons, including a mistrust in the accuracy of the tests [5, 6]. Provider strategies to overcome these challenges could include testing guidelines, as well as continuous training and monitoring [27, 29, 40] and the use of SMS messaging to improve both use of tests and compliance to test guidelines [41]. In addition, parallel efforts could be implemented such as incentive schemes, including bundling free ACT medicines with provider wholesale purchases of subsidized tests, to help promote provider uptake, distribution and increase profit margins [34, 42]. Such strategies and approaches may be adapted and refined according to each country’s context and regulatory framework(s).

## Conclusion

The results from this paper have shown that significant strides have been made in the availability of testing, especially in public health facilities, most notably due to an increase in the procurement of RDT. In the public sector, universal coverage of confirmatory diagnosis has almost been achieved in many countries and most confirmatory malaria tests are administered through this sector. However, stock-outs and procurement challenges must be continually monitored to ensure constant supply and uptake of RDT. In contrast, persistent gaps still remain in the private sector, with availability lagging behind the public sector, especially among pharmacies and drug stores, where most anti-malarials are distributed. This may be attributed to national regulations prohibiting the provision of malaria testing in these outlets, but also to RDT price barriers, particularly for children, which serve as a disincentive to test prior to treatment. These issues have the potential to impact both malaria control efforts and prospects for elimination, however several private sector strategies may provide innovative solutions for maximizing testing services across a range of contexts.

## Additional files

**Additional file 1.** Sample ACTwatch questionnaire.

**Additional file 2.** Percentage of anti-malarial-stocking community health workers and private sector general retailers and itinerant drug vendors with malaria RDT available on the day of the survey over time.

**Additional file 3.** Median private sector price for malaria microscopy, RDT, and pre-packaged quality-assured ACT treatment, across outlet types.

## Abbreviations

ACT: artemisinin-based combination therapy
AL: artemether–lumefantrine
ADDO: accredited drug dispensing outlets
AETD: adult equivalent treatment dose
ASAQ: artesunate–amodiaquine
CI: confidence interval
CHW: community health workers
DRC: Democratic Republic of the Congo
DFID: Department for International Development
DHIS2: District Health Information Software
DRC: Democratic Republic of Congo
IQR: interquartile range
RDT: rapid diagnostic test
N: number
N/A: not applicable
PPMV: proprietary patent medicine vendors
PSI: Population Services International
QAACT: quality-assured artemisinin combination therapy
SSA: sub-Saharan Africa
USD: US Dollar
WHO: World Health Organization
