# Supplementary material for: Testing times: trends in availability, price, and market share of malaria diagnostics in the public and private healthcare sector across eight sub-Saharan African countries from 2009 to 2015
Source: Malar J. 2017 May 19;16:205. doi: 10.1186/s12936-017-1829-5 (PMC5438573; doi:10.1186/s12936-017-1829-5)
Supplement: Supplementary file 1 — Additional file 1. Sample ACTwatch questionnaire. [file 12936_2017_1829_MOESM1_ESM.doc]

| **ACTwatch Outlet Survey UGANDA 2015** | | | | |
| --- | --- | --- | --- | --- |
|  | | | | |
| **Section 1: Census Information** | | | | |
| ***Interviewer completes this section for all outlets.*** | | | | |
| **Outlet ID: Interviewer-District- County-Sub-county-Outlet ID** [___|___]-[___|___]-[___|___|___]-[___|___|___|___]-[___|___|___] | | | | |
| C1. Today’s date (dd/mm/yyyy) | | | [___|___]-[___|___]-[_2_|_0_|_1_|_5_] | |
| C2. Interviewer’s name [_______________________________________________] | | | C2a. Interviewer’s code [___|___] | |
| C3. District [________________________________________________________] | | | C3a. District code [___|___] | |
| C4. County [_________________________________________________________] | | | C4a. County code [___|___|___] | |
| C5. Sub-county [______________________________________________________] | | | C5a. Sub-county code[___|___|___|___] | |
| C6. Name of outlet ***If no name, record “no name” or owner’s name***  [__________________________________________________________________] | | | C6a. Outlet code [___|___|___] | |
| C7. Type of Outlet  01 National Referral Hospital  02 Regional Referral Hospital  03 District / General Hospital  04 Health Centre IV – County  05 Health Centre III – Sub-county  06 Health Centre II – Parish    07 Community Medicine Distributor | 08 Pharmacy  09 Drug shop / Drug store  10 Private hospital  11 Private clinic / domiciliary / midwife  12 NGO/Mission hospital  13 NGO/Mission clinic | 14 Private diagnostics lab (*lab only)*  15 NGO/Mission diagnostics lab  (*lab only*)  96 Other ***(specify)***  [_______________________________] | | [___|___] |
| C8. Is this area part of the booster sample? 1 = Yes 0 = No | | | | [___] |

Hello, my name is __________, I work on behalf of the Programme for Accessible Health, Communication and Education, PACE. We are conducting a study on the availability of antimalarial medicines and diagnostic testing services. The results will be used to improve the availability of appropriate antimalarial treatment in Uganda. I would like to ask you a few questions to see if you could be part of the survey.

**Section 2: Screening & Eligibility**

| S1. Do you have any medicines in stock today?  1 = Yes ***Go to S3***  0 = No | [___] |
| --- | --- |
| S2. Are there any medicines that are out of stock today, but that you stocked in the **past 3 months?**  1 = Yes ***Go to S4***  0 = No ***Go to S5***  8 = Don’t know ***Go to S5*** | [___] |
| S3. Do you have any antimalarial medicines in stock today?  1 = Yes ***Provide information sheet & gain consent. Record start time in C9.***  ***Proceed to Section 3: Antimalarial Audit.***  0 = No Verify with prompt card. ***Go to S4*** | [___] |
| S4. Are there any antimalarial medicines that are out of stock today, but that you stocked in the **past 3 months?**  1 = Yes ***Provide information sheet & gain consent. Record start time in C9. Proceed to A16.***  0 = No ***Verify with prompt card. Go to S5***  8 = Don’t know ***Verify with prompt card. Go to S5*** | [___] |
| S5. Are you offering any diagnostic services or selling any diagnostic tests here today?  1 = Yes ***Go to S6***  0 = No ***Verify with prompt card. Record details in C9 then complete Sec 7: ORS & Zinc then***  ***Sec X: Ending Interview*** | [___] |
| S6. Are any of these services or tests for suspected malaria?  1 = Yes ***Provide information sheet & gain consent. Record start time in C9.  Proceed to Section 4: Diagnostic Audit***  0 = No ***Verify with prompt card. Record details in C9 then complete Sec 7: ORS & Zinc and***  ***Sec X: Ending Interview*** | [___] |

***Before proceeding to the full interview ensure you have given the respondent a study information sheet, explained the study and obtained informed consent***

C9. Result of Visit(s)

| Date  (dd/mm/yy) | **Visit 1** | **Visit 2** | **Visit 3** | | |
| --- | --- | --- | --- | --- | --- |
|  | [___\|___]-[___\|___]-[_1_\|_5_] | [___\|___]-[___\|___]-[_1_\|_5_] | [___\|___]-[___\|___]-[_1_\|_5_] | | |
| Time started ***(in 24hr clock)*** | [___|___]:[___|___] | [___|___]:[___|___] | [___|___]:[___|___] | | |
| Time completed ***(in 24hr clock)*** | [___|___]:[___|___] | [___|___]:[___|___] | [___|___]:[___|___] | | |
| Result | [___|___] | [___|___] | [___|___] | | |
|  | 01 = Outlet eligible & survey completed ***go to E1*** | | | | |
|  | 02 = Outlet ineligible: does not meet any screening criteria ***go to E1*** | | | | |
|  | 03 = Interview interrupted **go to C11** | | | | |
|  | 04 = Respondent not available/time not convenient **go to C11** | | | | |
|  | 05 = Outlet not open at the time **go to C11** | | | | |
|  | 06 = Outlet closed permanently **go to E1** | | | | |
|  | 96 = Other **(specify)**:[_________________________________________________________] | | | | |
|  | 97 = Refused **go to C10** | | | | |
| C10. If the provider refused, why?  1 = Client load ***Ask respondent for a time they would prefer to be interviewed and note in C11***  2 = Thinks it’s an inspection / nervous about license ***go to E1***  3 = Not interested ***go to E1***  6 = Other (specify):[___________________________________________________________________]  7 = Refuses to give reason ***go to E1*** | | | | [___] | |
| C11. ***Use this space to record call back details. If it is not possible to complete the interview at another time, go to E1.*** | | | | | |
| **Section 7: ORS, Zinc & Amoxicillin: *Read to the provider:*** I have just a few questions for you about availability of treatments for diarrhea and pneumonia | | | | | |
| ORS1. Do you have any oral rehydration salts, also known as ORS in stock today? ***Verify with prompt card.***  1 = Yes  0 = No | | | | | [___] |
| ORS2. Do you have any zinc tablets for treatment of diarrhea in children in stock today? ***Verify with prompt card.***  1 = Yes  0 = No ***Go to AB1*** | | | | | [___] |
| ORS3. Which strength of zinc tablets for treatment of diarrhea in children do you have in stock today?  ***Read list, circle ALL that apply*** 10mg  20mg  Other(specify )***:***[______________________________________] | | | | | A  B  C |
| ORS4. Do you have any ORS that is packaged together with zinc treatment for diarrhea in children in stock today?  ***Verify with prompt card***  1 = Yes  0 = No | | | | | [___] |
| AB1. Do you have any antibiotics in stock today?  1 = Yes  0 = No | | | | | [___] |
| AB2. Do you have any amoxicillin dispersible tablets, also known as Amox DT?  ***Show prompt card. Interviewer ask to see the product and verify that it is dispersible amoxicillin***  1 = Yes  0 = No ***Go to C9 – Results of visit*** | | | | | [___] |
| AB3. Which strength of amoxicillin dispersible tablets (Amox DT) do you have in stock today?  ***Read list, circle ALL that apply***  125mg  250mg  Other(specify )***:***[______________________________________] | | | | | A  B  C |

| **Section X: Ending the interview** | |
| --- | --- |
| E1. Name of interviewee:  [________________________________________________________]  *5 = Not applicable, no respondent; 7 = Refused* | |
| E2. Physical address or location identifiers of outlet (not PO box)***(Give detailed description that will help supervisor to find the outlet)*** | E3. Telephone number  [___|___|___|___|___|___|___|___|___|___]  *9999999995 = N/A: no respondent or has no telephone*  *9999999997 = Refused* |
| E4.Latitude:  [__] - **[___|___] .** [___|___|___|___|___|___|___|___] | E5.Longitude:  [_**E**_] - **[___|___] .** [___|___|___|___|___|___|___|___] |
| E7. **Additional observations by interviewer (if any)** | |

***THANK THE PROVIDER AND END INTERVIEW***

| **Section 3: Antimalarial Audit** |
| --- |

| ***A0. Read to the provider:***  Can you please show us the full range of antimalarials that you currently have in stock? Do you currently have any of the following?  ***Prompt entire list using antimalarial prompt card; No response to be recorded.***   - Artemether lumefantrine, such as *Lonart, Artefan, Lumartem, Coartem, Lumaren* - Artesunate amodiaquine, such as *DUAC, Coarsucam, Winthrop* - Other artemisinin combination therapies, such as *Arco* - Artemether monotherapies, such as *Larither, Artemether Rtitas, Artemedine, Artenam, Romether* - Artesunate monotherapies, such as *Plasmotrim, Artesun, G-Sunate* - Chloroquine, such as *Sugaquin, Mediquine, Kam quin, Bioquin, Renequin, Maxaquin, Oroquin* - SP, such as *Fansidar, Malaren, Kamsidar, Agosidar, Neosidar* - Quinine, such as *Quinas, Quine, Quinfer* - Amodiaquine, such as *Amobin* - Mefloquine, such as *Mephaquin, Meflotas* - Syrups or suspensions, such as *Quinine-K, Quinimix ,Requin, Ago-quinine ,Co-malartem suspension,* *Ago-CQ* - Injectables, such as *Rogoquin, Artemether, Quinax, Larither, Kwinil* - Granules or powders, such as *Artequin, Artesun*   ***If the outlet has no antimalarials in stock cross-check screening results then proceed to question A16.*** |
| --- |

***Proceed to the antimalarial audit. Different antimalarial audit sheets will be used to record the antimalarial information based on the dosage form of the medicine.***

***Separate the antimalarials into two piles:***

- ***The first pile should contain all the antimalarials in the form of tablets, suppositories, or granules.
  Use the Tablets, Suppositories & Granules Drug Audit Sheet to record these.***
- ***The second pile should contain all the antimalarials in any form other than tablets, suppositories or granules. Use the Non-Tablet Drug Audit Sheet to record these.***

***If additional audit sheets are used, add these sheets after the ones provided and staple the questionnaire again.***

***All pages should be in order before you move onto the next outlet.***

***Number each drug by assigning a Product Number (starting from 1 for TSG drugs and again from 1 for NT drugs).***

***Number each audit sheet used in the spaces provided at the bottom of the page.***

***ADDITIONAL NOTES ON THE SUB-OUTLET CODE***

***In all outlets, complete the Sub-Outlet Code (as well as the Product Number) for each drug audited. These codes are listed below.***

| ***SUB-OUTLET CODES*** | |
| --- | --- |
| X | ALL outlets that have only ONE dispensing/distribution point for medicines/diagnostics |
| A | Outpatient department / dispensary/Main pharmacy (if used by all patients) |
| B | Adult outpatient department / adult dispensary / adult clinic |
| C | Child outpatient department / child dispensary / child clinic |
| D | Antenatal / maternity clinic/MCH |
| E | ART / HIV/AIDS clinic |
| G | Private dispensing unit within a public health facility |
| L | Laboratory ***(for RDT audit)*** |
| Z | Other ***(specify the type in the space for audit comments –TSG 15 or NT 15)*** |

| **Sub-outlet code**  [_____]  _______  **Product number**  [__|__] | [__|__]  [__|__]  [__|__] | **1. Generic name** | | | | | **2. Strength**  **[__|__|__].**[__]mg  **[__|__|__].**[__]mg  **[__|__|__].**[__]mg | | **2a. Is this base strength?**  [__]  1 = Yes  [__] 0 = No  8 = Don’t know  [__]  ***If no, specify salt:***  [________________________] | | | | **3. Dosage form/formulation**  1 = Tablet  2 = Suppository  3 = Granule  [___] | **4. Brand name**  *(Include weight and age information)* | | |
| --- | --- | --- | --- | --- | --- | --- | --- | --- | --- | --- | --- | --- | --- | --- | --- | --- |
|  | [__\|__] | | | | | |  |  |  |  |  |  |  |  |  |  |
| **5. Manufacturer** | | | **6. Country of manufacture** | | | **7. Package size**  There are a total of  [___|___|___|___] tablets/ suppositories/ granule sachets in each:  1 = Package  2 = Pot/tin  [___] | | **8. Is product a fixed-dose combination (FDC)**  1 = Yes  0 = No  8 = Don’t   know  [___] | | | **9. Does product have the Green leaf logo?**  1 = Yes  0 = No  8 = Don’t   know  [___] | **10. Amount sold/distributed in the last 7 days to individual consumers** (*Record # of packages / tins described in* Q7 *OR record the total # of tablets / suppositories / granule packs sold*)  This outlet sold [___|___|___] **packages/ tins** in the last 7 days    **OR**  This outlet sold [___|___|___] **tablets/ suppositories or granule sachets** in the last 7 days  ***Not applicable = 995; Refused = 997; Don’t know = 998*** | | | | **11. Stocked out at any point in the past 3 months?**  1 = Yes  0 = No  8 = Don’t   know  [___] |
|  |  |  | [__\|__\|__] | | |  |  |  |  |  |  |  |  |  |  |  |
| **12. Retail selling price**  [___|___|___]  **tablets, suppositories or granule sachets** cost an individual customer  **[___|___|___|___|___]**  USH | | | | **13. Wholesale purchase price**  For the outlet’s most recent wholesale purchase  [___|___|___|___]  **tablets, suppositories or granule sachets** cost  **[___|___|___|___|___|___]**  USH | | | | | | **14. Why do you stock this medicine [SHOW PRODUCT]?**  ***Do not read list***.  ***Circle ALL responses given***  Free supply A  Profitable B  Recommended by the government C  Low price D  Customer demand or preference E  Positive brand reputation F  Often prescribed by doctors G  Most effective for treating malaria H  Don’t know X  Other Z  ***specify*** [_________________________________] | | | | | **15. Comments** | |
| ***Free = 00000***  ***Refused = 99997 Don’t know = 99998*** | | | | | ***Free = 000000***  ***Refused = 999997***  ***Don’t know = 999998*** | | | | |  |  |  |  |  |  |  |

Tablet Audit Sheet [__|__] of [__|__]

| **Sub-outlet code**  [_____]  _______  **Product number**  [__|__] | [__|__]  [__|__]  [__|__] | **1. Generic name** | | | | | **2. Strength**  **[__|__|__].**[__]mg  **[__|__|__].**[__]mg  **[__|__|__].**[__]mg | | **2a. Is this base strength?**  [__]  1 = Yes  [__] 0 = No  8 = Don’t know  [__]  ***If no, specify salt:***  [________________________] | | | | **3. Dosage form/formulation**  1 = Tablet  2 = Suppository  3 = Granule  [___] | **4. Brand name**  *(Include weight and age information)* | | |
| --- | --- | --- | --- | --- | --- | --- | --- | --- | --- | --- | --- | --- | --- | --- | --- | --- |
|  | [__\|__] | | | | | |  |  |  |  |  |  |  |  |  |  |
| **5. Manufacturer** | | | **6. Country of manufacture** | | | **7. Package size**  There are a total of  [___|___|___|___] tablets/ suppositories/ granule sachets in each:  1 = Package  2 = Pot/tin  [___] | | **8. Is product a fixed-dose combination (FDC)**  1 = Yes  0 = No  8 = Don’t   know  [___] | | | **9. Does product have the Green leaf logo?**  1 = Yes  0 = No  8 = Don’t   know  [___] | **10. Amount sold/distributed in the last 7 days to individual consumers** (*Record # of packages / tins described in* Q7 *OR record the total # of tablets / suppositories / granule packs sold*)  This outlet sold [___|___|___] **packages/ tins** in the last 7 days    **OR**  This outlet sold [___|___|___] **tablets/ suppositories or granule sachets** in the last 7 days  ***Not applicable = 995; Refused = 997; Don’t know = 998*** | | | | **11. Stocked out at any point in the past 3 months?**  1 = Yes  0 = No  8 = Don’t   know  [___] |
|  |  |  | [__\|__\|__] | | |  |  |  |  |  |  |  |  |  |  |  |
| **12. Retail selling price**  [___|___|___]  **tablets, suppositories or granule sachets** cost an individual customer  **[___|___|___|___|___]**  USH | | | | **13. Wholesale purchase price**  For the outlet’s most recent wholesale purchase  [___|___|___|___]  **tablets, suppositories or granule sachets** cost  **[___|___|___|___|___|___]**  USH | | | | | | **14. Why do you stock this medicine [SHOW PRODUCT]?**  ***Do not read list***.  ***Circle ALL responses given***  Free supply A  Profitable B  Recommended by the government C  Low price D  Customer demand or preference E  Positive brand reputation F  Often prescribed by doctors G  Most effective for treating malaria H  Don’t know X  Other Z  ***specify*** [_________________________________] | | | | | **15. Comments** | |
| ***Free = 00000***  ***Refused = 99997 Don’t know = 99998*** | | | | | ***Free = 000000***  ***Refused = 999997***  ***Don’t know = 999998*** | | | | |  |  |  |  |  |  |  |

***Tablet Audit Sheet [__|__] of [__|__]***

| **Sub-outlet code**  [_____]  _______  **Product number**  [__|__] | [__|__]  [__|__]  [__|__] | **1. Generic name** | | | | **2. Strength**  **[__|__|__|__].**[__]mg/**[__|__|__] .**[__]mL  **[__|__|__|__].**[__]mg/**[__|__|__] .**[__]mL  **[__|__|__|__].**[__]mg/**[__|__|__] .**[__]mL  **(*Note: no mL recorded for powder injection*)** | | | | **2a. Is this base strength?**  [__]  1 = Yes  [__] 0 = No  8 = Don’t know  [__]  ***If no, specify salt:***  [______________________] | | | **3. Dosage form/formulation**  1 = Syrup  2 = Suspension  3=Liquid injection  4 =Powder injection  5 = Drops  6 = Other ***(specify)*** [___________]  [___] | |
| --- | --- | --- | --- | --- | --- | --- | --- | --- | --- | --- | --- | --- | --- | --- |
|  | [__\|__] | | | | |  |  |  |  |  |  |  |  |  |
| **4. Brand name**  *(Include weight and age information)* | | | **5. Manufacturer** | | **6. Country of manufacture** | | | **7. Package size**  There are a total of  **[___|___|___|___].**[__] mL  (or mg for powder injections) in each:  1 = Bottle  2 = Ampoule/vial  [___] | **9. Does this product have the Green leaf logo?**  1 = Yes  0 = No  8 = Don’t   know  [___] | | **10. Amount sold/ distributed in the last 7 days to individual consumers**  This outlet sold  [___|___|___|___] **bottles, ampoules or vials** in the  last 7 days  ***Refused = 9997;***  ***Don’t know = 9998*** | | | **11. Stocked out at any point in the past 3 months?**  1 = Yes  0 = No  8 = Don’t   know  [___] |
|  |  |  |  |  | [__\|__\|__] | | |  |  |  |  |  |  |  |
| **12. Retail selling price**  [___|___|___]  **bottles ampoules or vials** cost an individual customer  **[___|___|___|___|___]** USH | | | | **13. Wholesale purchase price**  For the outlet’s most recent wholesale purchase:  [___|___|___|___]  **bottles, ampoules or vials** cost  **[___|___|___|___|___|___]** USH | | | **14. Why do you stock this medicine [SHOW PRODUCT]?**  ***Do not read list***.  ***Circle ALL responses given***  Free supply A  Profitable B  Recommended by the government C  Low price D  Customer demand or preference E  Positive brand reputation F  Often prescribed by doctors G  Most effective for treating malaria H  Don’t know X  Other Z  ***specify*** [_________________________________] | | | | | **15. Comments** | | |
| ***Free = 00000***  ***Refused = 99997***  ***Don’t know = 99998*** | | | | ***Free = 000000***  ***Refused = 999997***  ***Don’t know = 999998*** | | |  |  |  |  |  |  |  |  |

Non-Tablet Audit Sheet [___|___] of [___|___]

| **Sub-outlet code**  [_____]  _______  **Product number**  [__|__] | [__|__]  [__|__]  [__|__] | **1. Generic name** | | | | **2. Strength**  **[__|__|__|__].**[__]mg/**[__|__|__] .**[__]mL  **[__|__|__|__].**[__]mg/**[__|__|__] .**[__]mL  **[__|__|__|__].**[__]mg/**[__|__|__] .**[__]mL  **(*Note: no mL recorded for powder injection*)** | | | | **2a. Is this base strength?**  [__]  1 = Yes  [__] 0 = No  8 = Don’t know  [__]  ***If no, specify salt:***  [______________________] | | | **3. Dosage form/formulation**  1 = Syrup  2 = Suspension  3=Liquid injection  4 =Powder injection  5 = Drops  6 = Other ***(specify)*** [___________]  [___] | |
| --- | --- | --- | --- | --- | --- | --- | --- | --- | --- | --- | --- | --- | --- | --- |
|  | [__\|__] | | | | |  |  |  |  |  |  |  |  |  |
| **4. Brand name**  *(Include weight and age information)* | | | **5. Manufacturer** | | **6. Country of manufacture** | | | **7. Package size**  There are a total of  **[___|___|___|___].**[__] mL  (or mg for powder injections) in each:  1 = Bottle  2 = Ampoule/vial  [___] | **9. Does this product have the Green leaf logo?**  1 = Yes  0 = No  8 = Don’t   know  [___] | | **10. Amount sold/ distributed in the last 7 days to individual consumers**  This outlet sold  [___|___|___|___] **bottles, ampoules or vials** in the  last 7 days  ***Refused = 9997;***  ***Don’t know = 9998*** | | | **11. Stocked out at any point in the past 3 months?**  1 = Yes  0 = No  8 = Don’t   know  [___] |
|  |  |  |  |  | [__\|__\|__] | | |  |  |  |  |  |  |  |
| **12. Retail selling price**  [___|___|___]  **bottles ampoules or vials** cost an individual customer  **[___|___|___|___]** KSH | | | | **13. Wholesale purchase price**  For the outlet’s most recent wholesale purchase:  [___|___|___|___]  **bottles, ampoules or vials** cost  **[___|___|___|___|___]**  KSH | | | **14. Why do you stock this medicine [SHOW PRODUCT]?**  ***Do not read list***.  ***Circle ALL responses given***  Free supply A  Profitable B  Recommended by the government C  Low price D  Customer demand or preference E  Positive brand reputation F  Often prescribed by doctors G  Most effective for treating malaria H  Don’t know X  Other Z  ***specify*** [_________________________________] | | | | | **15. Comments** | | |
| ***Free = 0000***  ***Refused = 9997***  ***Don’t know = 9998*** | | | | ***Free = 00000***  ***Refused = 99997***  ***Don’t know = 99998*** | | |  |  |  |  |  |  |  |  |

Non-Tablet Audit Sheet [___|___] of [___|___]

**Antimalarials recently in stock**

| A16. Are there any antimalarial medicines that are out of stock today, but that you stocked in the  past **3 months?**  1 = Yes ***go to A17***  0 = No ***go to Section 4: Diagnostic Audit***  8 = Don’t know ***go to Section 4: Diagnostic Audit*** | [___] |
| --- | --- |
| A17. What are the names of the treatments that are out of stock?  ***Will accept generic or brand names. Record one medicine per line.***  1 = Yes, ***specify***  [_______________________________________] [_______________________________________]  [_______________________________________] [_______________________________________]  [_______________________________________] [_______________________________________]  [_______________________________________] [_______________________________________]  [_______________________________________] [_______________________________________]  0 = No, provider can’t remember | [___] |

***Interviewer: Go to Section 4: Diagnostic Audit***

| **Section 4: Diagnostic Audit**  *This section is about availability of malaria blood testing. Completing the questions may require speaking with more than 1 staff member at the outlet. If the respondent does not know the answer to a question in this section, ask to speak with another staff member who can provide the information.* |
| --- |

| D1. Does this outlet/facility have disposable gloves available today for staff to use when seeing customers/patients?  1 = Yes  0 = No  8 = Don’t know | [___] |
| --- | --- |
| D2. Does this outlet/facility have a sharps container, also called a sharps disposal box or safety box, available today for staff to use?  1 = Yes  0 = No  8 = Don’t know | [___] |
| D3. Is malaria microscopic testing available here today?  1 = Yes  0 = No **g*o to D7*** | [___] |
| D4. How many people were tested for **malaria** at this facility/outlet **using microscopy** within the past 7 days?  **997 = Refused; 998 = Don’t know** | [___|___|___] |
| D5.  What is the total cost for a microscopic test for malaria for an adult: **[___|___|___|___|___]**  USH  ***Free = 00000; NA =99995; Refused = 99997; Don’t know=99998*** | |
| D6.  What is the total cost for a microscopic test for malaria for a child under five: **[___|___|___|___|___]**  USH  ***Free = 00000; NA = 99995; Refused = 99997; Don’t know=99998*** | |
| D7. Malaria rapid diagnostic tests, also called RDTs, are small, individually wrapped blood tests that are able to quickly diagnose whether a person has malaria. ***Show RDT images in prompt card***  Are malaria RDTs available here today?  1 = Yes  0 = No  ***go to D9***  Don’t know ***ask to speak with a respondent who has this information*** | [___] |
| D8. Please show us the full range of RDTs that you currently have in stock. Do you currently have any of the following?  ***Read entire list; No response to be recorded.***   - *SD Bioline, Wondfo One Step, Nova test, AStel P.f* - *First Response, ParaCheck, Maleriscan, CTK on site rapid test* | |

***Proceed to the RDT audit.***

***If additional audit sheets are used, add these sheets after the ones provided and staple the questionnaire again. All pages should be in order before you move onto the next outlet.***

***Number each RDT by assigning a Product Number.***

***Number each audit sheet used in the spaces provided at the bottom of the page.***

***Complete the Sub-outlet Code as well as the Product Number for each RDT audited.***

***Sub-outlet codes are listed on page 4.***

| **Sub-outlet code**  [_____]  **Product number**  [__|__] | **1. Brand name** | | **2. Antigen test**  *(circle ALL that apply)*  HRP2 **A**  pLDH **B**  Aldolase **C**  Not indicated **Z** | | **3. Parasite species**  *(circle ALL that apply)*  Pf **A**  Pv **B**  Po **C**  pm **D**  pan **E**  vom/Pvom **F**  Other **G**  **Specify** [__________________]  Not indicated **Z** | | **4. Manufacturer** | | **5. Country of Manufacture** | **5b. Product Catalogue Number** | | **6. Lot Number** | **6b. Is this a self test kit, with each test kit co-packaged with its own buffer, pipette and lancet?**  1 = Yes  0 = No  8 = Don’t know  [___] | |
| --- | --- | --- | --- | --- | --- | --- | --- | --- | --- | --- | --- | --- | --- | --- |
| **13. Number of tests sold/ distributed /used in the last 7 days to individual consumers**  *(Record total # of tests)*  This outlet sold or distributed  [___|___|___|___] **tests** in the last 7 days  ***Refused = 9997; Don’t know=9998*** | | **14. Has this test been stocked out at any point in the past 3 months?**  1 = Yes  0 = No  8 = Don’t know  [___] | | **15a. Do you or other staff use this brand of RDT to test clients here at this facility/outlet?**  1 = Yes  0 = No ***go to 16a***  8 = Don’t know ***go to 16a***  [___]    **15b. If yes, what is the total cost for an adult to have a test conducted with this RDT, including RDT cost and service fee?**  **[___|___|___|___|___]**  USH  **15c. If yes, what is the total cost for a child under the age of five to have a test conducted with this RDT, including RDT cost and service fee?**  **[___|___|___|___|___]**  USH | | **16a. Does this facility/outlet provide this brand of RDT for clients to take away for testing somewhere else?**  1 = Yes  0 = No ***go to 17***  8 = Don’t know ***go to 17***  [___]    **16b. If yes, what is cost of this RDT for an adult?**  **[___|___|___|___|___]**  USH  **16c. If yes, what is the cost of this RDT for a child under the age of five?**  **[___|___|___|___|___]**  USH | | **17. Wholesale purchase price**  For the outlet’s most recent wholesale purchase:  [___|___|___|___] **tests**  cost  **[___|___|___|___|___|___]**  USH  ***Free = 000000***  ***NA = 999995***  ***Refused = 999997***  ***Don’t know=999998*** | | | **18.**  **Why do you stock this RDT [SHOW RDT]?**  ***Do not read list***  ***Circle ALL responses given***  Free supply A  Profitable B    Recommended by the government C  Low price D  Customer demand or preference E  Positive brand reputation F  Don’t know X  Other Z  ***specify*** [_______________________________] | | | **13. Comment** |
|  |  |  |  | ***Free = 00000; NA = 99995; Refused = 99997; Don’t know=99998*** | | | |  |  |  |  |  |  |  |

RDT Audit Sheet [___|___] of [___|___]

**RDT stock outs**

| D9. Are there any malaria RDTs that are out of stock today, but that you stocked in the past **3 months**?  1 = Yes  0 = No ***go to D11***  8 = Don’t know ***go to D11*** | [___] |
| --- | --- |
| D10. What are the brand names of the malaria RDTs that are out of stock?  ***Record one brand per line.***  1 = Yes, ***specify***  [____________________________________________________________________________]  [____________________________________________________________________________]  [____________________________________________________________________________]  0 = No, provider can’t remember | [___] |
| D11. Does this facility/outlet **provide medicines or prescription** for medicines?  1 = Yes ***go to Section 5: Provider Module***  0 = No ***check that S1 is no and S2 is no (the outlet has no medicines/had no medicines recently) and that C7 is 14 or 15***. ***Go to Section 7: ORS and ZINC and then Go to Section 6: Audit Tracking Sheet.*** | [___] |

| **Section 5: Provider Module**  ***This section is for the senior-most staff member who is responsible for providing treatment, prescriptions or medicines to clients/patients.*** |
| --- |

| P1. Do your responsibilities at this outlet/facility include: providing prescriptions, treatment, or medicines to clients?  1 = Yes  No ***ask to speak with the senior-most person at the outlet with 1 or more of these***  ***responsibilities.*** | | |___] |
| --- | --- | --- |
| P2. For how many years have you worked in this outlet/facility? **If less than 1 year, enter 01** | | [___|___] |
| P3. What age are you today? ***Write age in years***  97 = Refused  98 = Don’t know | [___|___] | |
| P4. ***Don’t read:*** Is respondent male or female?  1 = Male  2 = Female | [___|___] | |
| P5. What is the highest level of education you completed?   1. = No formal education 2. = Some primary school 3. = Completed primary school 4. = Some secondary school 5. = Completed secondary school 6. = Some university/college 7. = Completed a university/college degree/diploma | [___] | |
| P6. Have you received any training in the last 12 months that included a component on malaria diagnosis, including malaria rapid diagnostic tests or microscopy?  ***Include pre-service training and stand-alone workshops.***    1 = Yes  0 = No  8 = Don’t know | | [___] |
| P7. Have you received any training in the last 12 months on the national treatment guidelines for malaria? ***Include pre-service training and stand-alone workshops.***    1 = Yes  0 = No  8 = Don’t know | | [___] |
| P8. Do you have any of the following **health qualifications**?  ***Read list***.  ***Record 1 for yes, 0 for no*** | |  |
| 1. Dispenser (diploma in Pharmacy) | | [___] |
| 1. Pharmacist (Degree in Pharmacy) | | [___] |
| 1. Medical doctor | | [___] |
| 1. Clinical Officer | | [___] |
| 1. Nurse / Nursing Officer | | [___] |
| 1. Midwife | | [___] |
| 1. Laboratory technician / Lab assistant | | [___] |
| 1. Pharmaceutical technologist | | [___] |
| 1. Pharmacy technician | | [___] |
| 1. Public Health Technician/Officer | | [___] |
| 1. Health Assistant, Medical Assistant / Nursing Assistant / Nursing Aid | | [___] |
| 1. Community Medicine Distributor/Village Health Team | | [___] |

| P9. **Not** including yourself, do any other people working in this outlet or facility have the following **health qualifications**? ***Read list***.  ***Record 1 for yes, 0 for no, 8 for don’t know*** |  |
| --- | --- |
| 1. Dispenser (Diploma in Pharmacy) | [___] |
| 1. Pharmacist (Degree in Pharmacy) | [___] |
| 1. Medical doctor | [___] |
| 1. Clinical Officer | [___] |
| 1. Nurse / Nursing Officer | [___] |
| 1. Midwife | [___] |
| 1. Laboratory technician / Lab assistant | [___] |
| 1. Pharmaceutical technologist | [___] |
| 1. Pharmacy technician | [___] |
| 1. Public Health Technician/Officer | [___] |
| 1. Health Assistant, Medical Assistant / Nursing Assistant / Nursing Aid | [___] |
| 1. Community Medicine Distributor/Village Health Team | [___] |

| ***Interviewer:*** For the following questions record the antimalarial brand name or generic name, and dosage form, in the spaces provided. Ask the provider to show you the medicine if it is in stock to verify the name and dosage form. |
| --- |

| P10. In your opinion, for treating uncomplicated malaria in adults, what is the most effective antimalarial medicine?  ***Ask the provider to show you the medicine if it is in stock.***   | **Generic or brand name** | **Dosage form/formulation** | | | | --- | --- | --- | --- | | 01 = Tablet  02 = Suppository  03 = Granule | 04 = Syrup  05 = Suspension  06 = IM/IV Injection  (liquid or powder) | 07 = Drops  95 = None specified  98 = Don’t know | | [______________________________________]  ***Don’t know = 98*** | [___|___] | | | |
| --- | --- | --- | --- | --- | --- | --- | --- | --- | --- | --- | --- |
|  |

| P11. In your opinion, for treating uncomplicated malaria in children under five, what is the most effective antimalarial medicine?  ***Ask the provider to show you the medicine if it is in stock.***   | **Generic or brand name** | **Dosage form/formulation** | | | | --- | --- | --- | --- | | 01 = Tablet  02 = Suppository  03 = Granule | 04 = Syrup  05 = Suspension  06 = IM/IV Injection  (liquid or powder) | 07 = Drops  95 = None specified  98 = Don’t know | | [_______________________________________]  ***Don’t know = 98*** | [___|___] | | | |
| --- | --- | --- | --- | --- | --- | --- | --- | --- | --- | --- | --- |
|  |

| P12. What antimalarial medicine for treating uncomplicated malaria in adults do you most often recommend to customers?  ***Ask the provider to show you the medicine if it is in stock.***   | **Generic or brand name** | **Dosage form/formulation** | | | | --- | --- | --- | --- | | 01 = Tablet  02 = Suppository  03 = Granule | 04 = Syrup  05 = Suspension  06 = IM/IV Injection (liquid or powder) | 07 = Drops  95 = None specified  98 = Don’t know | | [______________________________________]  ***Don’t know = 98*** | [___|___] | | | |
| --- | --- | --- | --- | --- | --- | --- | --- | --- | --- | --- | --- |
|  |

| P13. What antimalarial medicine for treating uncomplicated malaria in children under five do you most often recommend to customers?  ***Ask the provider to show you the medicine if it is in stock.***   | **Generic or brand name** | **Dosage form/formulation** | | | | --- | --- | --- | --- | | 01 = Tablet  02 = Suppository  03 = Granule | 04 = Syrup  05 = Suspension  06 = IM/IV Injection (liquid or powder) | 07 = Drops  95 = None specified  98 = Don’t know | | [______________________________________]  ***Don’t know = 98*** | [___|___] | | | |
| --- | --- | --- | --- | --- | --- | --- | --- | --- | --- | --- | --- |
|  |

| P14. In your opinion, for treating severe malaria in children under five, what is the most effective antimalarial medicine?  ***Ask the provider to show you the medicine if it is in stock.***   | **Generic or brand name** | **Dosage form/formulation** | | | | --- | --- | --- | --- | | 01 = Tablet  02 = Suppository  03 = Granule | 04 = Syrup  05 = Suspension  06 = IM/IV Injection (liquid or powder) | 07 = Drops  95 = None specified  98 = Don’t know | | [_____________________________________]  ***Don’t know = 98*** | [___|___] | | | |
| --- | --- | --- | --- | --- | --- | --- | --- | --- | --- | --- | --- |
|  |

| P15. What antimalarial medicine for treating severe malaria in children under five do you most often recommend to customers?  ***Ask the provider to show you the medicine if it is in stock.***   | **Generic or brand name** | **Dosage form/formulation** | | | | --- | --- | --- | --- | | 01 = Tablet  02 = Suppository  03 = Granule | 04 = Syrup  05 = Suspension  06 = IM/IV Injection (liquid or powder) | 07 = Drops  95 = None specified  98 = Don’t know | | [_____________________________________]  ***Don’t know = 98*** | [___|___] | | | |
| --- | --- | --- | --- | --- | --- | --- | --- | --- | --- | --- | --- |
|  |

| P16. Please name the first line medicine recommended by the government to treat uncomplicated malaria.  ***Do not read list. Only one response allowed.***  01 = Artemether Lumefantrine *(Lonart, Artefan, Lumartem, Coartem*) …………………………… ***Go to P17***  02 = ACT................................................................................................................................ ***Go to P17***  03 = ACTm............................................................................................................................. ***Go to P17***  04 = Artesunate Amodiaquine *(DUAC, Coarsucam, Winthrop)*  05 = Dihydroartemisinin Piperaquine *(Duo-cotecxin, P-alaxin)*  06 = Amodiaquine  07 = Artemether*(Artenam, Paluther, Artesiane, Larither)*  08 = Artemisinin  09 = Artesunate *(Artesun, Larinate, Plasmotrim)* ***Go to P19***  10 = Chloroquine *(Sugarquin)*  11 = Quinine  12 = Sulfadoxine Pyrimethamine *(Fansidar, SP, Orodar, Ekelfin, Metakelfin)*  96 = Other *specify*: [______________________________________________]  98 = Don’t know | [___|___] |
| --- | --- |

| P17. Please explain the government recommended treatment regimen for this drug for an adult (60kg)  ***What is the dosage formulation?***   | 01 = Tablet  02 = Suppository  03 = Granule | 04 = Syrup  05 = Suspension  06 = Injection (IV/IM) | 07 = Drop  95 = None specified  96 = Not applicable  98 = Don’t know | | --- | --- | --- |   ***If provider responded not tablet then skip to p18***  ***Read the following 3 questions to the provider***   1. How many tablets should they take at a time? 2. How many times per day? 3. Over how many days?   ***If respondent has the medicine available use the package to complete the table below.***  ***If the medicine is not available ask respondent to identify from prompt card.***  ***If identification not possible, ask respondent to recall medicine details.***   |  | **Generic name** | **Strength in mg** | **Brand name** | | --- | --- | --- | --- | | [__|__] | ______________________________  ______________________________  ______________________________ | [__|__|__].[__]mg  [__|__|__].[__]mg  [__|__|__].[__]mg |  | | [__|__]  [__|__] | |  | |  | [___|___] | ***Don’t know=999.8*** | | **Manufacturer** | | Is this drug a **fixed-dose combination**  1 = Yes  0 = No  8 = Don’t know  [___] | | | **Don’t know = 98** | | | [___|___]  [___|___]**.**[___|___]  [___|___]  [___|___]  ***Don’t know = 98*** |
| --- | --- | --- | --- | --- | --- | --- | --- | --- | --- | --- | --- | --- | --- | --- | --- | --- | --- | --- | --- | --- | --- | --- | --- |
|  |  |

| P18. Please explain the government recommended treatment regimen for this drug for a 2-year old child (10kg) ***Read the following 3 questions to the provider***  ***What is the dosage formulation?***   | 01 = Tablet  02 = Suppository  03 = Granule | 04 = Syrup  05 = Suspension  06 = Injection (IV/IM) | 07 = Drop  95 = None specified  96 = Not applicable  98 = Don’t know | | --- | --- | --- |   ***If provider responded not tablet then skip to p19***  ***Read the following 3 questions to the provider***   1. How many tablets should they take at a time? 2. How many times per day? 3. Over how many days?   ***If respondent has the medicine available use the package to complete the table below.***  ***If the medicine is not available ask respondent to identify from prompt card.***  ***If identification not possible, ask respondent to recall medicine details.***   |  | **Generic name** | **Strength in mg** | **Brand name** | | --- | --- | --- | --- | | [__|__] | ___________________________  ___________________________  ___________________________ | [__|__|__].[__]mg  [__|__|__].[__]mg  [__|__|__].[__]mg |  | | [__|__] | | [__|__] | |  | [___|___] | ***Don’t know=999.8*** | | **Manufacturer** | | Is this drug a **fixed-dose combination**  1 = Yes  0 = No  8 = Don’t know  [___] | | | **Don’t know = 98** | | | [___|___]  [___|___]**.**[___|___]  [___|___]  [___|___]  ***Don’t know = 98*** |
| --- | --- | --- | --- | --- | --- | --- | --- | --- | --- | --- | --- | --- | --- | --- | --- | --- | --- | --- | --- | --- | --- | --- | --- |
|  | |

| P19. Please name the medicine recommended by the government to treat severe malaria.  ***Do not read list. Only one response allowed.***  01 = Artesunate *(Artesun, Larinate, Plasmotrim)* ………………………………………………………………. ***Go to P20***  02 = Artemether *(Artenam, Paluther, Artesiane, Larither)* ..................................................... ***Go to P20***  03 = Quinine............................................................................................................................ ***Go to P20***  04 = ACT/ACTm  05= Artemether Lumefantrine *(Lonart, Artefan, Lumartem, Coartem)*  06 = Artesunate Amodiaquine *(DUAC, Coarsucam, Winthrop)*  07 = Dihydroartemisinin Piperaquine *(Duo-cotecxin, P-alaxin)* ***Go to P21***  08 = Chloroquine (Sugarquin)  09 = Sulfadoxine Pyrimethamine *(Fansidar, SP, Orodar, Ekelfin, Metakelfin)*  96 = Other (specify): [ _________________________________________]  98 = Don’t know | | | [___|___] | |
| --- | --- | --- | --- | --- |
| P20. What is the drug formulation for the recommended medicine by the government to treat severe malaria? ***Do not read dosage form options*** | | | [___|___] | |
| 01 = Tablet  02 = Suppository  03 = Granule | 04 = Syrup  05 = Suspension  06 = IM/IV Injection (Liquid or powder) | 07 = Drops  95 = None specified  98 = Don’t know |
| P21. Malaria rapid diagnostic tests, also called RDTs, are small, individually wrapped blood tests that are able to quickly diagnose whether a person has malaria. ***Show RDT images in prompt card***  Have you ever seen or heard of malaria RDTs?  1 = Yes ***Go to P22***  0 = No  ***Go to P29***  8 = Don’t know ***Go to P29*** | | | | [___] |
| P22. Have you ever tested a client for malaria using an RDT?  1 = Yes  0 = No  8 = Don’t know | | | | [___] |
| P23. Would you ever recommend a patient/customer take an antimalarial if a blood test using a rapid diagnostic test produced a negative test result for malaria? **Read list. Record only one response.**  1 = Yes, Sometimes  2 = Yes, Always  3 = No, Never ***go to P29***  8 = Don’t know ***go to P29*** | | | | [___] |
| P24. Under what circumstances would you recommend a patient/customer take an antimalarial following a negative RDT test for malaria? ***Do not read list****.* ***Prompt “anything else” until the respondent is finished.***  ***Circle ALL responses given*** | | | |  |
| When they have signs/symptoms of malaria | | | | A |
| When they ask for antimalarial treatment | | | | B |
| When they are a child | | | | C |
| When they are an adult | | | | D |
| When they are a pregnant woman | | | | E |
| When I do not trust/believe the test | | | | F |
| When I know the patient/customer | | | | G |
| Other (specify) [_______________________________________________________] | | | | X |

| P29. What are the danger signs of severe illness in a child under 5?  ***Do not read list****.* ***Prompt “anything else” until the respondent is finished.***  ***Circle ALL responses given*** |  | |
| --- | --- | --- |
| Unable to drink /unable to breastfeed | A | |
| Vomits everything | B | |
| Convulsions | C | |
| Lethargic or unconscious | D | |
| Don’t know | Z | |
| Other (specify) [__________________________________________________________] | X | |
| P30. What would you do if a 2-year old child was brought to this outlet with the danger signs of **severe** illness?  ***Do not read list****.* ***Only one response allowed.***  01 = Seek advice/help from someone in this facility  02 = Treat the child in this facility  03 = Refer to a health facility (clinic, hospital) with or without treating here  04 = Refer to a non health facility outlet (not a clinic or hospital) with or without treating here  05 = Send them away/home without medicine  06 = Send them away/home with medicine  96 = Other (specify): [__________________________________________________________]  98 = Don’t know | [___|___] |  |

***Complete the audit sheet tracker on the next page then follow the instructions for ending the interview.***

| **Section 6: Audit Tracking Sheet** |
| --- |

| T1. Were there any antimalarial TABLETS/SUPPOSITORIES/GRANULES in stock at this outlet?  1 = Yes  0 = No ***go to T4***  8 = Don’t know ***go to T4*** | [___] |
| --- | --- |
| T2. Total number of TABLET/SUPPOSITORY/GRANULE audit sheets completed | [___|___] |
| T3. Did you complete audit sheet information for all available TABLETS/SUPPOSITORIES/GRANULES*?*  1 = Yes, audit complete  0 = No, audit not complete | [___] |

| T4. Were there any antimalarial NON TABLETS (Syrups, suspensions, Injectables) in stock at this outlet?  1 = Yes  0 = No ***go to T7***  8 = Don’t know ***go to T7*** | [___] |
| --- | --- |
| T5. Total number of NON-TABLET audit sheets completed | [___|___] |
| T6. Did you complete audit sheet information for all available NON-TABLETS*?*  1 = Yes, audit complete  0 = No, audit not complete | [___] |

| T7. Were there any RDTs in stock at this outlet?  1 = Yes  0 = No ***go to T10***  8 = Don’t know ***go to T10*** | [___] |
| --- | --- |
| T8. Total number of RDT audit sheets completed | [___|___] |
| T9. Did you complete audit sheet information for all available RDT*?*  1 = Yes, audit complete  0 = No, audit not complete | [___] |

| T10. COMMENTS: Reason for incomplete audit sheets (if response is no to T3, T6, or T9): |
| --- |

***Be sure to complete Section 7: ORS, Zinc & Amoxicillin, then to C9 and record the final status of the interview and time completed and proceed to Section X: Ending the Interview.***
