# Supplementary material for: Testing times: trends in availability, price, and market share of malaria diagnostics in the public and private healthcare sector across eight sub-Saharan African countries from 2009 to 2015
Source: Malar J. 2017 May 19;16:205. doi: 10.1186/s12936-017-1829-5 (PMC5438573; doi:10.1186/s12936-017-1829-5)
Supplement: Supplementary file 2 — Additional file 2. Percentage of anti-malarial-stocking community health workers and private sector general retailers and itinerant drug vendors with malaria RDT available on the day of the survey over time. [file 12936_2017_1829_MOESM2_ESM.docx]

**Additional file 2: Percentage of anti-malarial-stocking community health workers and private sector general retailers and itinerant drug vendors with malaria RDT available on the day of the survey over time**

|  | **Community Health Workers** | | | **General Retailers and Itinerant Drug Vendors** | |
| --- | --- | --- | --- | --- | --- |
|  | N | | % Outlets stocking mRDT  (95% CI) | N | % Outlets stocking mRDT  (95% CI) |
| **West & Central Africa** | | | | | |
| Benin |  | |  |  |  |
| 2011 | 49 | | 0.0 | 743 | 0.0 |
| 2014 | 60 | | 12.5 (2.0, 50.5) | 1266 | 0.1 (<0.1, 0.4) |
| Kinshasa (DRC) |  | |  |  |  |
| 2009 | 0 | | - | 0 | - |
| 2013 | 0 | | - | 1 | 0.0 |
| 2015 | 0 | | - | 0 | - |
| Katanga  (DRC) |  | |  |  |  |
| 2013 | 0 | | - | 15 | 4.0 (0.5, 26.8) |
| 2015 | 0 | | - | 0 | - |
| Nigeria |  | |  |  |  |
| 2009 | 15 | | 0.0 | 103 | 0.0 |
| 2011 | 10 | | 0.0 | 114 | 0.0 |
| 2013 | 7 | | 8.4 (1.1, 44.3) | 88 | 0.0 |
| 2015 | 9 | | 8.7 (1.0, 46.7) | 132 | 0.0 |
| **East Africa** | | | | | |
| Kenya | |  |  |  |  |
| 2010 | 63 | | 0.0 | 1094 | 0.0 |
| 2011 | 3 | | 0.0 | 575 | 0.0 |
| 2014 | 51 | | 26.4 (11.4, 49.9) | 403 | 0.0 |
| Tanzania |  | |  |  |  |
| 2010 | 2 | | 0.0 | 90 | 0.0 |
| 2011 | 0 | | - | 22 | 4.4 (0.4, 34.2) |
| 2014 | 0 | | - | 21 | 8.5 (2.3, 27.2) |
| Uganda |  | |  |  |  |
| 2010 | 34 | | 11.8 (1.0, 65.3) | 35 | 0.0 |
| 2011 | 107 | | 70.2 (36.4, 90.6) | 24 | 0.0 |
| 2013 | 836 | | 53.4 (29.3, 76.0) | 2 | 0.0 |
| 2015 | 900 | | 58.2 (35.5, 77.9) | 0 | - |
| **Southern Africa** | | | | | |
| Madagascar | |  |  |  |  |
| 2010 | 51 | | 12.5 (2.3, 47.0) | 1609 | 0.0 |
| 2011 | 98 | | 54.2 (39.9, 67.8) | 1513 | 0.0 |
| 2013 | 334 | | 88.8 (82.6, 93.0) | 335 | 0.5 (0.1, 3.5) |
| 2015 | 165 | | 72.8 (59.4, 83.1) | 314 | 0.1 (<0.1, 0.4) |
| Zambia |  | |  |  |  |
| 2009 | 0 | | - | 56 | 0.0 |
| 2011 | 0 | | - | 147 | 0.5 (0.1, 3.0) |
| 2014 | 16 | | 83.2 (49.3, 96.2) | 135 | 0.1 (<0.1, 0.5) |
|  | | | | | |
