# Supplementary material for: Testing times: trends in availability, price, and market share of malaria diagnostics in the public and private healthcare sector across eight sub-Saharan African countries from 2009 to 2015
Source: Malar J. 2017 May 19;16:205. doi: 10.1186/s12936-017-1829-5 (PMC5438573; doi:10.1186/s12936-017-1829-5)
Supplement: Supplementary file 3 — Additional file 3. Median private sector price for malaria microscopy, RDT, and pre-packaged quality-assured ACT treatment, across outlet types. [file 12936_2017_1829_MOESM3_ESM.docx]

**Additional file 3: Median private sector price for malaria microscopy, mRDT, and pre-packaged quality-assured ACT treatment, across outlet types^**

|  | Adult | | | Child | | |
| --- | --- | --- | --- | --- | --- | --- |
|  | Microscopy  Median [IQR] (N) | mRDT  Median [IQR] (N) | QA AL Adult  Median [IQR] (N)** | Microscopy  Median [IQR] (N) | mRDT  Median [IQR] (N) | QA AL Pediatric*  Median [IQR] (N) |
| **Benin 2014** |  |  |  |  |  |  |
| Private Facility | 3.09 [2.47-4.12] (54) | NS  (15) | 2.06  [1.24-2.47] (28) | 3.09  [2.47-4.12] (55) | NS  (15) | NS  (15) |
| Pharmacy/Drug Store | -  (0) | -  (0) | 6.02  [3.86-6.75] (556) | -  (0) | -  (0) | 1.44  [1.44-1.44] (161) |
| Total Private Sector | 3.09 [2.47-4.74] (55) | NS  (17) | 1.65[1.44-3.61] (761) | 3.09  [2.47-4.74] (56) | NS  (17) | 0.62  [0.41-0.82] (419) |
| **Kinshasa (DRC) 2015** |  |  |  |  |  |  |
| Private Facility | 1.10  [1.10-1.10](198) | 1.10 [0.55-1.10] (73) | 0 [0-0] (26) | 1.10  [1.10-1.10] (198) | 1.10  [0.55-1.10] (73) | 0 [0-0] (21) - |
| Pharmacy/Drug Store | 1.10 [0.77-1.10] (10) | 0.55 [0.00-1.10] (4) | 2.19 [1.63-2.19] (14) | 1.10 [0.55-1.10] (10) | 0.55 [0.00-1.10] (4) | 1.64 [1.10-1.64] (13) |
| Total Private Sector | 1.10 [1.10-1.10] (208) | 1.10 [0.55-1.10] (77) | 0 [0-1.10]  (40) | 1.10 [1.10-1.10] (208) | 1.10 [0.55-1.10] (77) | 0 [0-1.10]  (34) |
| **Katanga (DRC) 2015** |  |  |  |  |  |  |
| Private Facility | 1.10 [0.55-2.19] (49) | 1.10 [0.00-1.10] (83) | 0 [0-0.55]  (39) | 1.10[0.55-1.64]  (49) | 1.10 [0.00-1.10] (83) | 0 [0-0.55]  (36) |
| Pharmacy/Drug Store | 1.64 [1.10-2.19] (6) | 1.10 [0.55-1.64] (38) | 0.66 [0.55-1.10] (90) | 1.10 [0.88-1.64] (6) | 1.10 [0.55-1.64] (38) | 0.55 [0.33-0.55] (68) |
| Total Private Sector | 1.10  [1.10-2.19] (55) | 1.10 [0.55-1.10] (121) | 0.55 [0.33-1.10] (129) | 1.10 [0.55-1.64] (55) | 1.10 [0.55-1.10] (121) | 0.44 [0.33-0.55] (104) |
| **Nigeria 2015** |  |  |  |  |  |  |
| Private Facility | 1.52 [1.52-2.03] (130) | 1.01 [1.01-2.54] (100) | 0.76 [0.51-1.78] (34) | 1.52 [1.52-2.03] (130) | 1.01 [1.01-2.54] (100) | 0.76 [0.51-1.01] (24) |
| Pharmacy/Drug Store | 5.07 [5.07-5.07] (7) | 0.51 [0.25-1.01] (172) | 1.52 [1.01-1.78] (1522) | 5.07 [5.07-5.07] (7) | 0.51 [0-1.01]  (164) | 0.76 [0.51-1.01] (1645) |
| Total Private Sector | 1.52 [1.52-2.03] (137) | 1.01 [1.01-1.52] (272) | 1.27 [1.01-1.78] (1599) | 1.52 [1.52-2.03] (137) | 1.01 [0.51-1.52] (264) | 0.76 [0.51-1.01] (1705) |
| **Kenya 2014** |  |  |  |  |  |  |
| Private Facility | 1.13 [0.56-1.13] (263) | 1.13 [1.13-1.69]  (134) | 1.58 [1.13-1.69]  (240) | 1.13 [0.56-1.13] (263) | 1.13 [1.13-1.69] (133) | 0.56 [0.56-1.13] (65) |
| Pharmacy/Drug Store | 1.13[1.13-1.13] (85) | 1.13[1.13-1.69] (114) | 1.13 [1.13-1.69] (912) | 1.13 [1.13-1.13] (85) | 1.13[1.13-1.13] (112) | 0.56 [0.56-0.90] (292) |
| Total Private Sector | 1.13 [0.56-1.13] (348) | 1.13 [1.13-1.69] (248) | 1.13 [1.13-1.69] (1194) | 1.13 [0.56-1.13] (348) | 1.13 [1.13-1.69] (245) | 0.56 [0.56-1.13] (363) |
| **Tanzania 2014** |  |  |  |  |  |  |
| Private Facility | 0.59 [0.59-0.59] (160) | 0.89 [0.59-1.18] (102) | 1.18 [0.89-1.48] (110) | 0.59 [0.59-0.59] (160) | 0.65 [0.59-1.18] (101) | 0.59 [0.30-0.89] (78) |
| Pharmacy/Drug Store | -  (11) | 0.89 [0.59-1.18] (66) | 1.18 [0.89-1.82] (1189) | -  (11) | 0.59 [0.59-1.18] (65) | 0.59 [0.30-0.59] (821) |
| Total Private Sector | 0.59 [0.59-0.59] (171) | 0.59 [0.59-1.18] (171) | 1.18 [0.89-1.82] (1308) | 0.59 [0.59-0.59] (171) | 0.59 [0.59-1.18] (169) | 0.59 [0.30-0.59] (906) |
| **Uganda 2015** |  |  |  |  |  |  |
| Private Facility | 0.97 [0.65-0.97] (409) | 0.97 [0.65-0.97] (513) | 1.62 [1.29-2.26] (662) | 0.97 [0.65-0.97] (410) | 0.97 [0.65-0.97] (514) | 0.65 [0.32-0.97] (86) |
| Pharmacy/Drug Store | 0.65 [0.48-0.97] (27) | 0.81 [0.65-0.97] (434) | 1.62 1.29-1.62] (1843) | 0.65 [0.48-0.97] (26) | 0.65 [0.65-0.97] (435) | 0.32 [0.32-0.48] (403) |
| Total Private Sector | 0.97 [0.65-0.97] (436) | 0.81 [0.65-0.97] (947) | 1.62 [1.29-1.94] (2505) | 0.81 [0.65-0.97] (436) | 0.81 [0.65-0.97] (949) | 0.39 [0.32-0.65] (489) |
| **Madagascar 2015** |  |  |  |  |  |  |
| Private Facility | 1.58 [0.95-2.52] (27) | 0.16 [0.00-0.32] (120) | 0.00 [0.00-0.00] (33) | 1.58 [0.95-2.52] (27) | 0.16 [0.00-0.32] (120) | 0.32 [0.00-0.32] (49) |
| Pharmacy/Drug Store | -  (0) | 0.32 [0.32-0.32] (31) | -  (10) | -  (0) | 0.32 [0.32-0.32] (31) | 0.19 [0.16-0.25] (55) |
| Total Private Sector | 1.58  [0.95-2.52] (27) | 0.19  [0.00-0.32] (152) | 0.19  [0.00-0.22] (43) | 1.58  [0.95-2.52] (27) | 0.19  [0.00-0.32] (152) | 0.19  [0.16-0.32] (107) |
| **Zambia 2014** |  |  |  |  |  |  |
| Private Facility | -  (11) | -  (16) | -  (7) | -  (11) | -  (15) | -  (4) |
| Pharmacy/Drug Store | -  (4) | 0.82 [0.82-1.64] (72) | 3.28 [1.64-4.92] (130) | -  (4) | 0.82 [0.82-1.64] (72) | 0.82 [0.49-2.46] (38) |
| Total Private Sector | -  (15) | 1.23 [0.82-3.28] (89) | 2.46 [1.64-4.92] (142) | -  (15) | 1.23 [0.82-3.28] (88) | 0.82 [0.49-1.97] (45) |
| ^ Inclusive of private for-profit health facilities and pharmacies/drug stores. General retailers and itinerant drug vendors contribute to overall private sector medians where they were found to have anti-malarials or malaria blood testing in stock, or anti-malarials in stock in the previous three months. However, N’s for mRDT and microscopy price estimates among the category of general retailer/itinernat durg vendor are well below 20 in all cases (most often N=0) and therefore are not shown here.  * Pre-packaged treatment for a 2-year old child  **In Kinshasa, Katanga and Madagscar this is Artesunate-amodiaquine | | | | | | |
